# Electrocatalytic Urea Synthesis From CO_2_ and N_2_/NO_3_^−^: Bridging Catalyst Activity, C–N Coupling Kinetics, and Industrial Applicability

**DOI:** 10.1002/advs.78592

**Published:** 2026-11-04

**Authors:** Fulin Dong, Ruihan Zhang, Huiyu Lou, Tianshi Gao, Guangjin Zhang, Menglei Yuan

**Affiliations:** ^1^ State Key Laboratory of Solidification Processing and School of Materials Science and Engineering Northwestern Polytechnical University Xi'an China; ^2^ Queen Mary University of London Engineering School Northwestern Polytechnical University Xi'an China; ^3^ CAS Key Laboratory of Green Process Engineering Institute of Process Engineering Chinese Academy of Sciences Beijing China

**Keywords:** C–N coupling, CO_2_ conversion, electrocatalysis, nitrate reduction, reaction mechanism, single‐atom catalysts, urea synthesis

## Abstract

Electrocatalytic urea synthesis enables the co‐conversion of CO_2_ and N_2_/NO_3_^−^ under ambient conditions and offers a sustainable alternative to conventional energy‐intensive processes. This review systematically summarizes the NO_3_^−^ + CO_2_ and N_2_ + CO_2_ systems in terms of feedstock properties, catalyst design, reaction mechanisms, performance optimization, and engineering applications. The NO_3_^−^ + CO_2_ system, benefiting from facile NO_3_^−^ activation and high aqueous accessibility, has achieved significant improvements in efficiency and selectivity through rate matching and side‐reaction suppression, with performance approaching practical viability. The N_2_ + CO_2_ system, though more sustainable in feedstock, remains constrained by the high N≡N bond energy and low solubility, with current research focusing on N_2_ activation and intermediate regulation, while performance still lags considerably. The two systems differ markedly in nitrogen activation pathways and regulatory mechanisms, corresponding to distinct developmental stages and application potentials. Finally, challenges and future directions are outlined in terms of catalyst design, mechanistic studies, reactor engineering, and standardized evaluation, providing guidance for the construction of efficient systems and their scale‐up applications.

## Graphical abstract

This review compares NO_3_^−^ + CO_2_ and N_2_ + CO_2_ electrocatalytic urea synthesis in feedstock properties, catalyst design, C–N coupling mechanisms, performance optimization, and engineering applicability. The two systems correspond to a near‐term deployable route and a long‐term green synthesis goal, respectively.

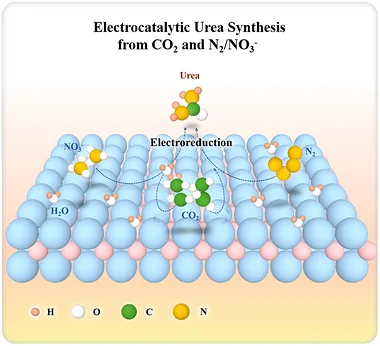

## 1 Introduction

Urea is not only one of the most widely used nitrogen fertilizers globally, but also an important bulk chemical feedstock. It finds extensive applications in agriculture, resin synthesis, pharmaceuticals, and the fine chemical industry [1, 2, 3]. Currently, the global annual demand for urea has exceeded 200 million tons, and its industrial production has long relied on the two‐step “ammonia synthesis–urea synthesis” process. During this process, N_2_ and H_2_ are first converted into NH_3_ via the Haber–Bosch process, followed by the further reaction of NH_3_ with CO_2_ under high temperature and high pressure to produce urea [4, 5]. Although this route has been industrialized on a large scale, it suffers from notable drawbacks: first, the Haber–Bosch process typically requires operation at 350–550°C and 150–350 bar, entailing high energy consumption and stringent equipment requirements [2, 6, 7]. Second, the hydrogen source is mainly derived from fossil resources such as natural gas reforming, resulting in a significant dependence of the entire process on fossil energy [7]. Third, the overall carbon footprint of the conventional urea industry is high, which is detrimental to the establishment of a green chemical system under the “dual carbon” goals [7, 8, 9]. Meanwhile, environmental issues arising from the imbalance in the carbon and nitrogen cycles have become increasingly prominent. On one hand, excessive agricultural fertilization, industrial wastewater discharge, and domestic sewage leakage have led to the continuous accumulation of nitrate in water bodies. Excessively high NO_3_^−^ concentrations not only disrupt aquatic ecological balance but also pose risks to drinking water safety and human health [10]. On the other hand, massive CO_2_ emissions have caused the greenhouse effect to intensify continuously and have become one of the core drivers of global climate change [11]. Therefore, how to synergistically convert nitrogen‐containing pollutants and low‐value or even harmful molecules such as CO_2_ into high‐value‐added chemicals has become an important research direction in the interdisciplinary fields of energy, environment, and catalysis [12].

Against this backdrop, electrocatalytic urea synthesis technology has attracted widespread attention. Driven by renewable electricity, this technology enables the synergistic activation of nitrogen‐ and carbon‐source molecules and the construction of C–N bonds under ambient temperature and pressure, offering advantages such as mild reaction conditions, broad feedstock availability, and compatibility with intermittent renewable energy sources like wind and solar power. More importantly, the electrocatalytic route is expected to directly utilize CO_2_ from industrial flue gas, NO_3_^−^ from wastewater, or N_2_ from the air as feedstocks, thereby breaking free from the dependence of conventional urea production on high temperature, high pressure, and fossil resources, and enabling a distributed, low‐carbon, and sustainable novel synthesis paradigm [13, 14].

Depending on the type of nitrogen source, current electrocatalytic urea synthesis can be mainly divided into two core systems: the NO_3_^−^ + CO_2_ system and the N_2_ + CO_2_ system [8, 15, 16]. The former employs nitrate as the nitrogen source, offering advantages such as high nitrogen‐source solubility, low activation difficulty, and relatively facile intermediate generation. Consequently, it has made rapid progress in Faradaic efficiency (FE), urea yield rate (UYR), and reactor scale‐up, and is currently the most actively researched route that is closest to practical application [17, 18, 19]. The latter, in contrast, directly employs N_2_ from the air as the nitrogen source, offering the most abundant feedstock and the greatest theoretical potential for “fully green synthesis.” However, due to the high bond energy of the N≡N bond, the difficulty of molecular polarization, and its low solubility in aqueous solutions, its adsorption activation, stabilization of nitrogen‐containing intermediates, and subsequent C–N coupling all face higher kinetic barriers [20, 21, 22]. As a result, its overall performance still lags considerably behind that of the NO_3_^−^ + CO_2_ system. Precisely for this reason, the two systems are not simply in a parallel relationship, but rather correspond respectively to a “near‐term deployable route for pollutant resource utilization” and a “long‐term worthy full‐green synthesis route.”

The research on electrocatalytic urea synthesis can be traced back to 1995, when Furuya et al. first achieved urea production via the co‐reduction of CO_2_ and NO_x_^−^ using a Cu‐loaded gas diffusion electrode [23, 24]. In recent years, significant progress has been achieved in electrocatalytic urea synthesis research. This has been driven by the development of novel catalytic systems such as single‐atom/dual‐atom catalysts, heterostructures, MOF/COF materials, and frustrated Lewis pairs, along with continuous advancements in in situ infrared spectroscopy, in situ XAS, and theoretical calculations [18, 25, 26, 27, 28]. For the NO_3_^−^ + CO_2_ system, the research focus has gradually shifted from the development of single active sites to the construction of bifunctional sites, modulation of interfacial electronic structures, and suppression of side reactions [18, 26]. For the N_2_ + CO_2_ system, the research emphasis has been centered on enhancing N_2_ activation, stabilizing nitrogen‐containing intermediates, and identifying C–N coupling pathways [29, 30]. Meanwhile, the reactor configuration has also gradually expanded from the conventional H‐cell to flow cells, gas diffusion electrodes, and membrane electrode assemblies, propelling this field from conceptual validation toward engineering exploration (Figure 1).

**FIGURE 1:**
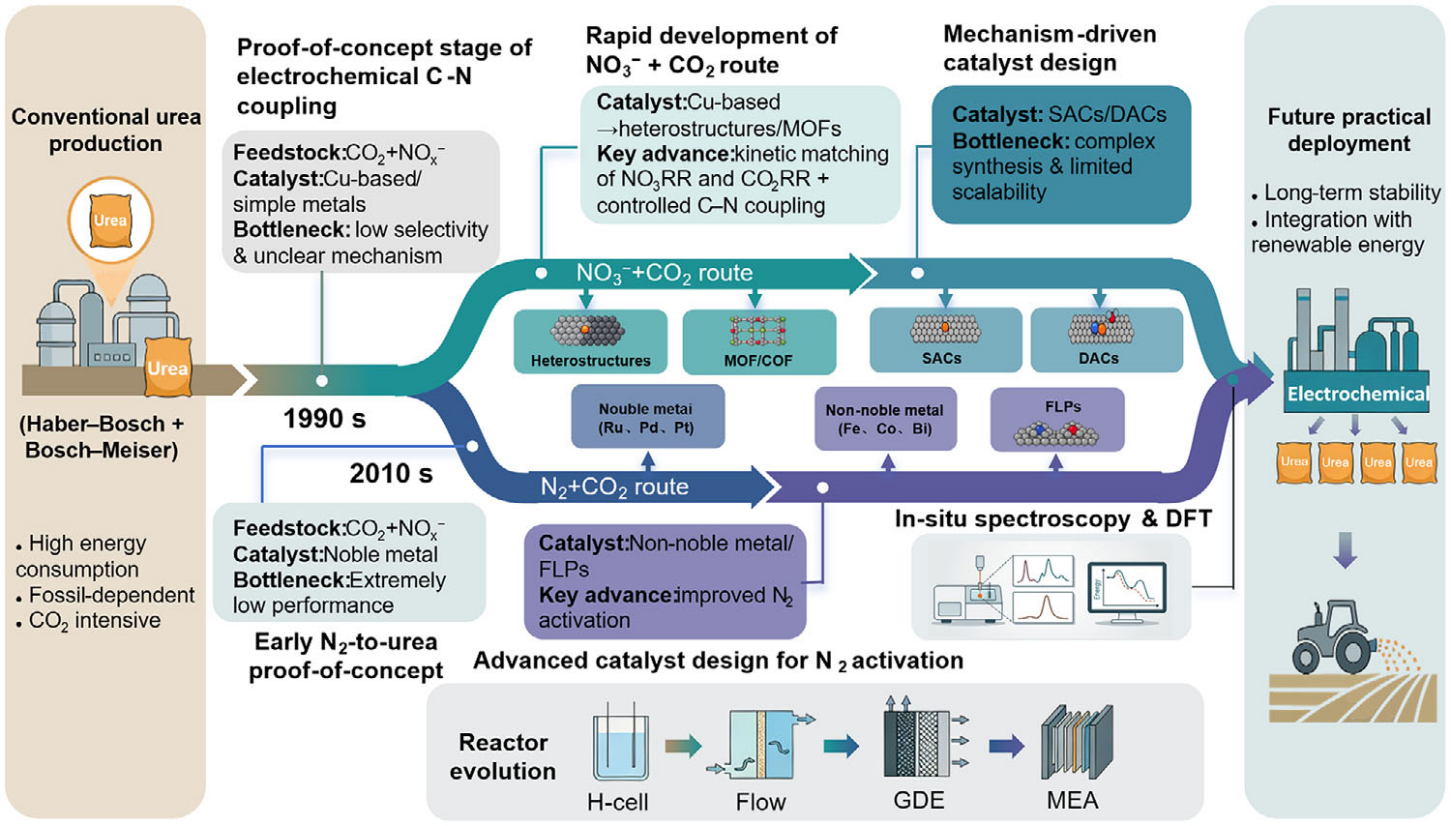
Evolution of electrocatalytic urea synthesis from conventional processes to mechanism‐driven catalyst and system design.

Despite the rapid development of this field, existing reviews have largely focused on a single nitrogen‐source system, specific catalyst categories, or a particular class of reaction mechanisms, lacking a systematic comparison between the two core routes of NO_3_^−^ + CO_2_ and N_2_ + CO_2_. In fact, these two systems differ significantly in terms of feedstock properties, catalyst design logic, key intermediate evolution, C–N coupling modes, performance upper limits, and application scenarios. A parallel review and comparative summary of the two systems would be more conducive to grasping the general principles and key bottlenecks in this field.

On this basis, this review takes the progressive chain of feedstock characteristics, catalyst design, reaction mechanisms, performance optimization, and engineering exploration as the main thread to systematically summarize the latest research progress on the NO_3_^−^ + CO_2_ and N_2_ + CO_2_ electrocatalytic urea synthesis systems. It emphasizes comparing their similarities and differences in active site construction, intermediate regulation, C–N coupling mechanisms, and technological maturity, while further discussing their applicable scenarios and future development directions. It is hoped that the summary presented in this review will provide a reference for the rational design of highly active, highly selective, and highly stable catalysts, as well as for the scale‐up application of electrocatalytic urea synthesis technology.

## 2 Electrocatalytic Urea Synthesis System of NO_3_^−^ + CO_2_

In the electrocatalytic urea synthesis system involving NO_3_^−^ and CO_2_, the different reduction onset potentials of NO_3_^−^ and CO_2_ lead to a kinetic mismatch between the CO_2_ reduction reaction (CO_2_RR) and the nitrate reduction reaction (NO_3_^−^RR). The extremely complex intermediate species generated during co‐electrolysis impose great challenges on the targeted synthesis of urea via C–N coupling, along with competition from side reactions such as the hydrogen evolution reaction (HER) [31]. The performance of this system depends on the synergistic catalytic capability of the catalyst toward the two reactions, the adsorption strength of intermediates, and the effectiveness of side‐reaction suppression. This section provides a review from three dimensions: catalyst design, reaction mechanism, and performance optimization.

### 2.1 Catalyst Design Strategies and Structure–Performance Relationships

Catalysts are central to regulating the synergy between NO_3_^−^RR and CO_2_RR, and the key to their design lies in constructing active sites and microenvironments that are compatible with both reactions, thereby achieving full‐process optimization encompassing NO_3_^−^ activation, CO_2_ conversion, and intermediate coupling. Specifically, it is necessary to precisely regulate the electronic states, spatial distribution, and reaction microenvironments of the active sites to simultaneously satisfy the requirements for efficient NO_3_^−^ reduction, targeted CO_2_ activation, and proximal coupling of reaction intermediates [32, 33].

#### 2.1.1 Active Site Engineering

The electronic states, coordination environments, and spatial distributions of active sites directly influence the adsorption and conversion efficiency of intermediates. Current research primarily focuses on the design and regulation of single‐atom, dual‐atom/multi‐atom, alloy/heterostructure, MOF/COF‐based, and metal‐free sites [34, 35, 36, 37, 38, 39, 40, 41, 42, 43, 44]. Each type of site is tailored through differentiated structural design to meet the synergistic requirements of CO_2_RR and NO_3_^−^RR.

##### 2.1.1.1 Single‐Atom Catalysts (SACs)

Single‐atom catalysts feature 100% atom utilization efficiency and precisely defined coordination environments, enabling the optimization of intermediate adsorption energies through tuning the types of central atoms and coordinating atoms [34, 35, 47]. For example, in the Ru_1_/CuO_x_ single‐atom catalyst, Ru atoms are anchored onto the oxygen vacancies (OVs) of CuO_x_ via a Ru–O_3_ configuration [17]. The Ru_1_–OV sites enable specific adsorption and reduction of NO_x_^−^ to the *NH_2_ intermediate, while the Cu–OV sites are responsible for adsorbing and activating CO_2_ to generate the *CO intermediate. The synergy between the two sites achieves a high FE of 86.7% for urea. The Cu–N_3_ single‐atom catalyst underwent in situ electrochemical reconstruction from copper single sites into dual sites (Figure 2a), which were restructured into an N_2_–Cu–Cu–N_2_ configuration under an applied electric field (Figure 2b–d), exhibiting remarkable performance for the electrocatalytic co‐reduction of NO_3_^−^ and CO_2_ to synthesize urea [45]. At −0.9 V versus RHE, it achieves a UYR of 19597.65 ± 1821.24 mg h^−1^ mg_Cu_^−1^ and a FE of 55.4 ± 1.89%. In addition, studies have demonstrated that Ti, V, Nb, Mo, and Hf–N_4_C catalysts simultaneously satisfy key criteria including thermodynamic stability, favorable adsorption of small molecules (Figure 2e–g), suppression of side reactions, and low energy barriers for hydrogenation and C–N coupling [46]. Electronic structure analysis reveals that moderate hybridization between metal d orbitals and N‐p orbitals both stabilizes intermediates and preserves catalytic reactivity (Figure 2h).

**FIGURE 2:**
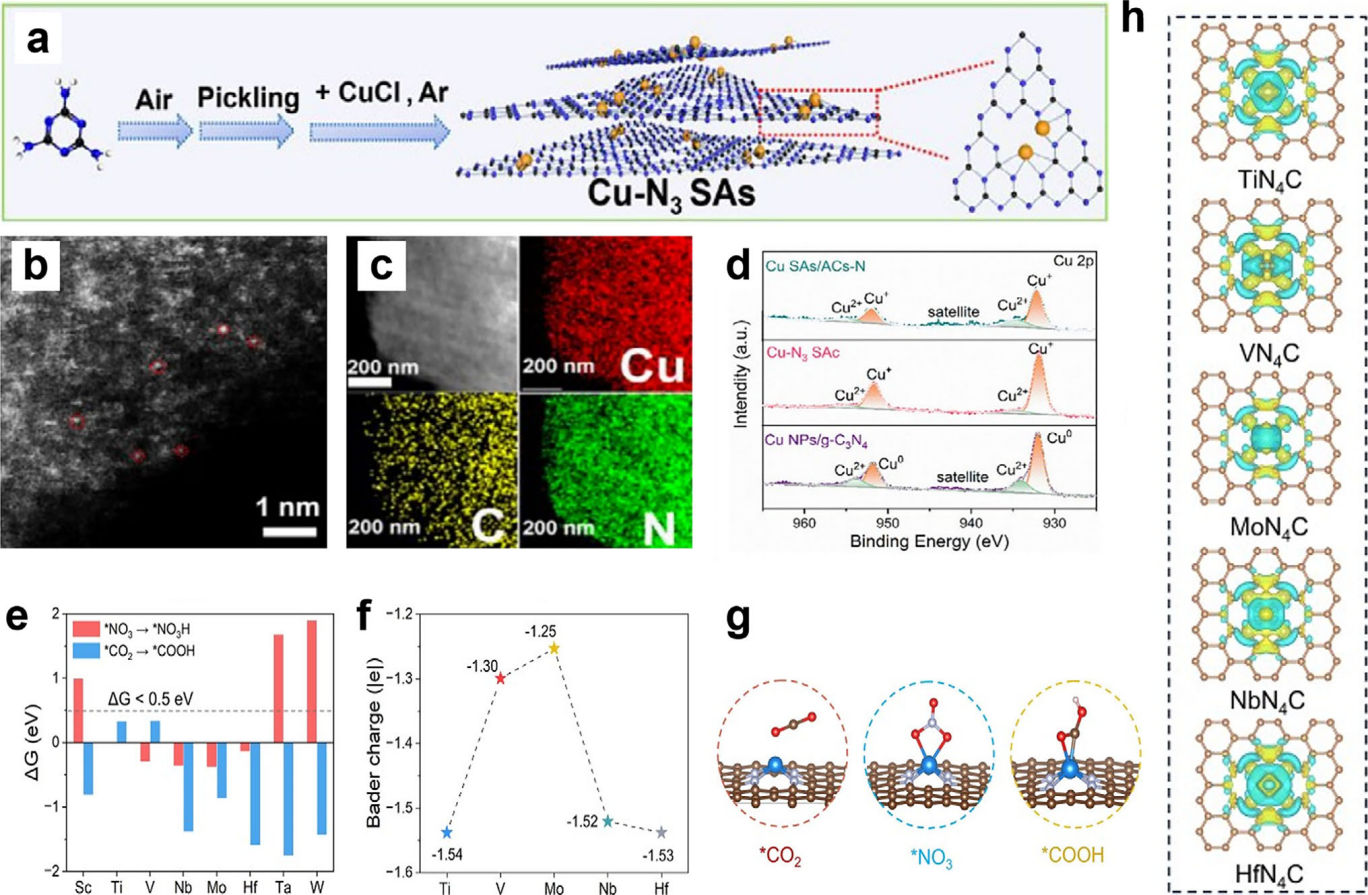
a) Scheme for the synthesis and characterization of Cu‐N_3_ SAs. b) Magnified HAADF‐STEM image of Cu‐N_3_ SAs, and c) element mapping images. d) Cu K‐edge XANES spectra of Cu‐N_3_ SAs and Cu SAs/ACs‐N. (Cu foil, Cu_2_O, and CuO as references). Reproduced with permission [45]. Copyright 2025, Wiley‐VCH. e) Gibbs free energy variations in the first‐step hydrogenation of NO_3_^−^ and CO_2_. f) Bader and h) differential charges of M atoms in the MN_4_C (M = Ti, V, Nb, Mo, and Hf). g) Lattice structure of *CO_2_, *NO_3_, and *COOH. Reproduced with permission [46]. Copyright 2025, Wiley‐VCH.

##### 2.1.1.2 Dual‐Atom/Multi‐Atom Catalysts

Dual‐atom catalysts (DACs) leverage the cross‐tuning effect between two metal atoms to separately accommodate the intermediate requirements of NO_3_^−^RR and CO_2_RR, thereby addressing the challenge that a single atomic site can hardly optimize both reactions simultaneously [36, 48, 49]. In the high‐density Ga–Y dual‐atom catalyst (Ga/Y–CNP), the Ga and Y sites synergistically complement each other to substantially lower the energy barriers [50]. The Ga site is responsible for activating CO_2_ to generate *CO, while the Y site reduces NO_3_^−^ to produce *NH_2_OH. At the adjacent Ga–Y atomic pair, the intermediates spontaneously couple to form urea, achieving a UYR of 41.9 mmol h^−1^ g^−1^ and exhibiting stable performance without decay over 21 consecutive cycles. The SrCo_0.39_Ru_0.61_O_3‐δ_ catalyst exhibits excellent catalytic activity and selectivity, with Co and Ru sites serving as the active centers for the formation of *CO and *NH_2_ intermediates, respectively [51]. The introduction of Co increases the number of oxygen vacancies and enhances the density of Ru 4d electronic states, which optimizes the adsorption strength of intermediates such as *NH_2_. The modulation of the electronic structures of Co and Ru sites by oxygen vacancies weakens the adsorption of *CO and *NH_2_ intermediates, while the strain imposed on the Co–Ru dual sites promotes the C–N coupling process between the two monomers and prevents their direct separation into byproducts. In addition, studies have shown that the spin‐polarized PdCuCo atomically ordered intermetallic compound, through the precise division of labor and synergistic interplay among Pd, Cu, and Co atoms, achieves exceptional performance [52]. Pd sites activate CO_2_ to generate *CO, Cu sites adsorb and reduce NO_3_^−^, and Co sites regulate the hydrogenation pathway of intermediates via spin polarization while suppressing side reactions. This catalyst achieves a high FE of 81% and a UYR of 227 mmol g_cat_^−1^ h^−1^, with electrochemical stability exceeding 260 h. This synergistic strategy of site division and electronic/strain/structural regulation exhibits general applicability in dual‐atom and multi‐atom catalysts, providing abundant design concepts for highly efficient urea electrosynthesis.

##### 2.1.1.3 Alloy and Heterostructure Catalysts

Alloys and heterostructures regulate the electronic states of active sites and the adsorption strength of intermediates through interfacial electron transfer and lattice strain effects, while simultaneously constructing multiple reaction domains to achieve division of labor and synergy [37, 38, 39, 55, 56, 57]. In the Sn/Cu_2_O heterostructure, Sn and Cu_2_O form a well‐defined heterointerface with interfacial electron transfer [58]. The Sn sites (with a d_10_ electronic configuration) can suppress the hydrogen evolution side reaction and assist CO_2_ activation, while the Cu^+^/Cu^0^ sites derived from Cu_2_O are responsible for the targeted reduction of NO_3_^−^. The synergy between the two sites promotes the C–N coupling of key intermediates. The crystalline Cu core/amorphous CuO_x_ shell hybrid nanowire heterojunction catalyst (C–Cu–A_4_) establishes a unique electrochemical–chemical cycling mechanism through electron transfer and oxygen vacancy regulation at the crystalline–amorphous heterointerface (Figure 3a–c) [53]. The process proceeds via initial electroreduction to generate Cu^0^ and oxygen vacancies, followed by oxygen vacancy‐mediated NO_3_^−^ activation to produce the nitrogen‐bonded *NO_2_ intermediate and concomitant oxidation of Cu^0^ to Cu^+^ (Figure 3d,e). Ultimately, Cu^+^ catalyzes the C–N coupling between *NO_2_ and CO_2_. This pathway reduces the electron transfer requirement from 16e^−^ to 12e^−^, bypassing the conventional rate‐limiting step, and achieves a record UYR of 0.89 mol h^−1^ g^−1^ with a stable structure during operation. The mixed‐valence intermetallic Cu_2_Sb alloy catalyst, prepared via wet impregnation combined with high‐temperature annealing under a H_2_/Ar atmosphere, features stable Cu^+^–Cu^2+^ dual sites within its crystal structure [59]. The Cu^+^ sites stabilize the *NO intermediate, while the Cu^2+^ sites promote *CO formation. The synergistic interplay between the two sites lowers the C–N coupling energy barrier to 0.23 eV, achieving a UYR of 22.9 mmol h^−1^ g^−1^ and a FE of 64.9% at −0.4 V versus RHE, along with long‐term operational stability exceeding 200 h. In addition, Wu et al. [60] developed a PdAuCuIrCo high‐entropy alloy featuring a disordered atomic arrangement that moderately weakens the adsorption strength of *NO_2_, preventing excessive stabilization of intermediates that would otherwise lead to exclusive reduction to NH_3_, while simultaneously promoting co‐adsorption with CO_2_. The introduction of Pd not only modulates the electron transfer efficiency but also reduces the energy barriers for C–N coupling and hydrogenation steps. Ultimately, this catalyst achieves a UYR of 52.43 mmol g^−1^ h^−1^ and a FE of 22.57% at −0.9 V versus RHE. This study provides a framework for the development of multi‐pathway electrocatalytic reactions.

**FIGURE 3:**
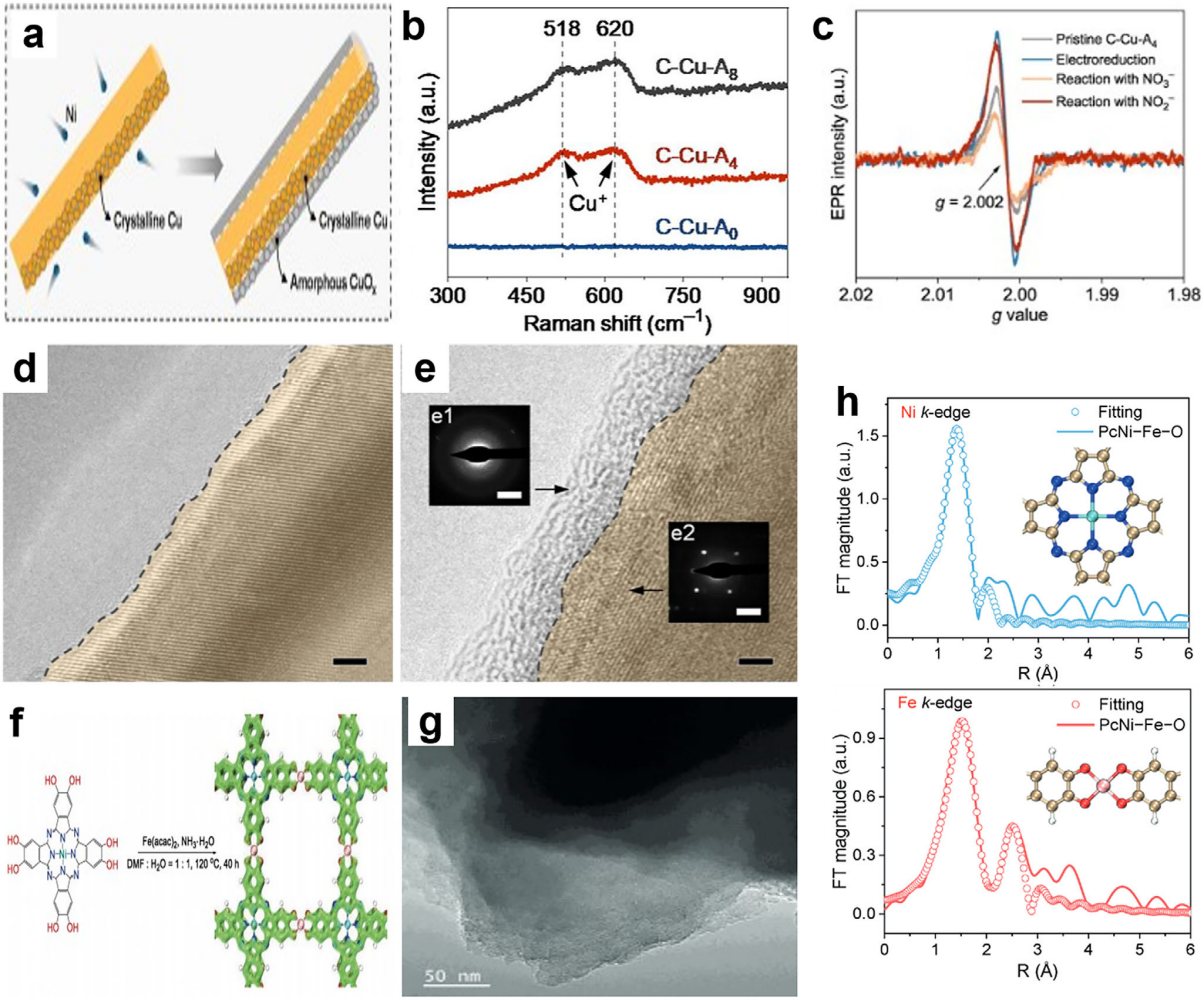
a) Schematic illustration of the synthetic process for crystalline−amorphous Cu‐based hybrids. b) Raman spectra of the C−Cu‐A_0_, C−Cu‐A_4_, and C−Cu‐A_8_ catalysts. c) EPR spectra of C−Cu‐A_4_: pristine (before electroreduction), after electroreduction, and after chemical reaction with NO_3_^−^/NO_2_^−^. HRTEM images of (d) crystalline C−Cu‐A_0_, e) C−Cu‐A_4_ with corresponding SAED patterns (insets). Reproduced with permission [53]. Copyright 2025, American Chemical Society. f) Illustration of the synthesis and structure of PcNi‐Fe‐O. The green isosurfaces represent the conjugated π‐electron density. g) TEM of PcNi‐Fe‐O. h) The EXAFS fitting curve of the Ni sites and Fe sites, respectively, in PcNi‐Fe‐O. Color codes: Fe, Pink; Ni, Cyan; C, Gold; O, Red; H, White. Reproduced with permission [54]. Copyright 2024, Wiley‐VCH.

##### 2.1.1.4 MOF/COF‐Based Catalysts

The porous structures of metal–organic frameworks (MOFs) and covalent organic frameworks (COFs) can enhance mass transfer, while their tunable coordination environments and pore sizes enable precise regulation of the spatial distribution and electronic states of active sites [40, 41, 42, 61, 62, 63]. The PCOF‐34‐Fe catalyst developed by Peng et al. [64] is the most representative example. Through modular assembly of 8‐connected porphyrin‐based stereoscopic nodes, a three‐dimensional entangled COF network was constructed, in which Fe atoms were coordinated with N to form sub‐nanometer diiron sites with an Fe–Fe distance of 8.8 Å, enabling direct visualization of the catalytic site array via low‐dose cryo‐electron microscopy (Low‐dose cryo‐EM). The dual sites can separately adsorb NO_3_^−^ and CO_2_, directionally guiding the migration and coupling of intermediates. In a commercial gas diffusion electrode (GDE)‐coupled system, the urea yield rate is enhanced to 135.6 mmol g_cat_^−1^ h^−1^ at −0.6 V, with a nitrogen selectivity of 92.4% and a carbon selectivity approaching 100%, and no significant performance decay is observed over 100 h of continuous operation (Figure 4c,d). Similarly, Liao et al. [54] designed a two‐dimensional dual‐active‐site MOF catalyst, PcNi–Fe–O, which was assembled via a solvothermal method by coordinating nickel phthalocyanine (NiPc) ligands with planar square‐planar FeO_4_ nodes, forming spatially separated dual active sites with appropriate inter‐site distances (Figure 3f–h). The FeO_4_ sites exclusively catalyze the reduction of NO_3_^−^ to NH_3_, while the NiPc sites activate CO_2_ and react with the *NH intermediate to form the key *NHCOOH intermediate, thereby circumventing the limitation of a single active site in balancing the adsorption of two types of intermediates. Meanwhile, the NH_3_ generated at the FeO_4_ sites can efficiently migrate to the NiPc sites and couple with *NHCOOH to complete the second C–N bond formation, significantly lowering the reaction energy barrier. This catalyst exhibits excellent performance in a neutral electrolyte, achieving a FE for urea of 54.1% at −0.6 V versus RHE with no performance decay over 20 h of continuous operation. Upon scaling the electrode area to 25 cm^2^ and operating for 8 h, 0.164 g of high‐purity urea can be produced, demonstrating clear potential for industrial application (Figure 4e,f).

**FIGURE 4:**
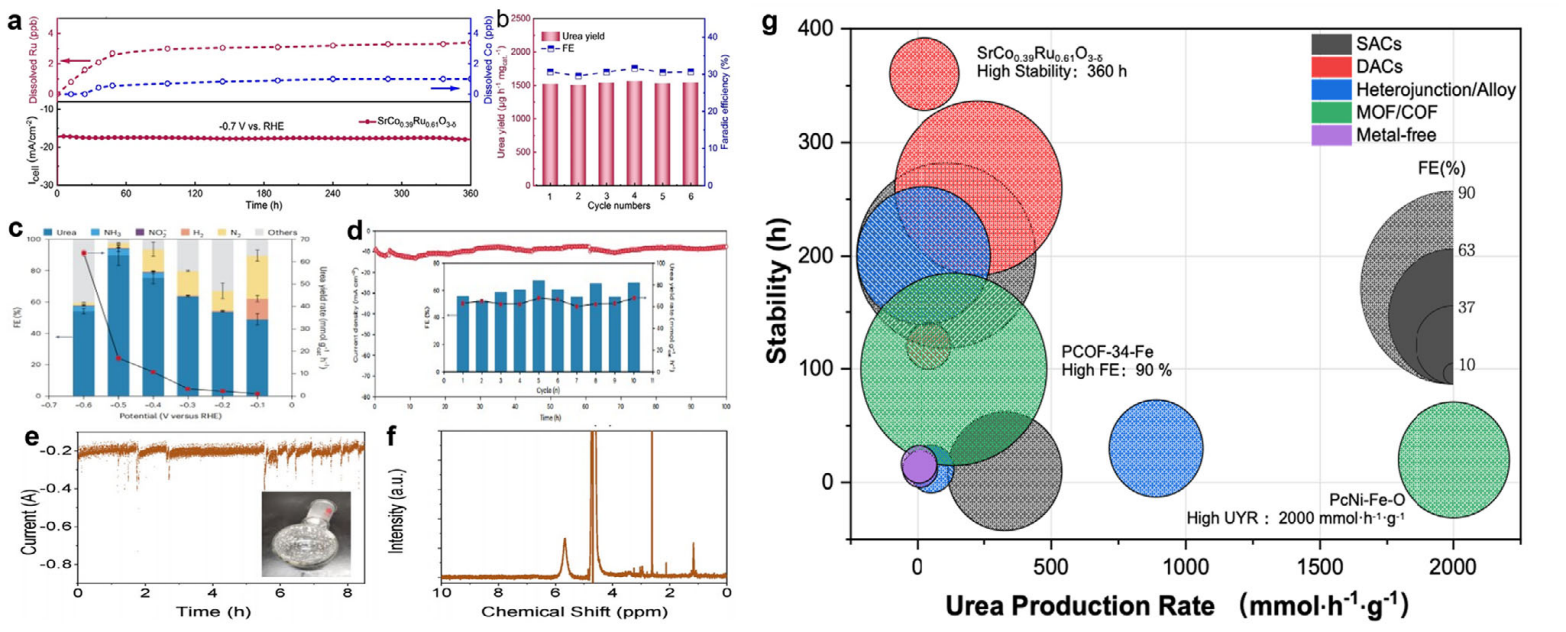
a) Chronoamperometric durability test at 0.7 V. b) The cyclic stability test of SrCo_0.39_Ru_0.61_O_3‐δ_. The error bars represent the standard deviation for three independent measurements. Reproduced with permission [51]. Copyright 2024, Wiley‐VCH. c) Potential‐dependent urea production rate and FE for PCOF‐34‐Fe. The error bars represent the s.d. of three independent experiments, and the data are presented as mean ± s.d. d) I–t curve recorded at −0.6 V. Inset: cycling stability. Reproduced with permission [64]. Copyright 2025, Springer Nature. e) Long‐term i‐t in a 25 cm^2^ flow‐cell device at 0.6 V vs. RHE of PcNi‐Fe‐O. f) 1 H NMR spectra of the urea product. Reproduced with permission [54]. Copyright 2024, Wiley‐VCH. g) Bubble charts showing the stability, yield, and Faraday efficiency of the above 13 types of catalysts.

##### 2.1.1.5 Metal‐Free Catalysts

Metal‐free catalysts employ carbon materials as the matrix, with active sites constructed through heteroatom doping (e.g., N, P, F). They combine the advantages of low cost, high stability, and environmental friendliness. Their catalytic activity originates from the charge transfer effect between dopant atoms and the carbon matrix, enabling precise regulation of intermediate adsorption and reaction pathways [43, 44, 65, 66, 67]. Wang et al. [68] developed an N‐doped carbon catalyst (N–C–100) via pyrolysis of a zinc‐coordinated polymer, which featured a high specific surface area of 1600 m^2^ g^−1^ with well‐defined division of labor among different N species. Pyridinic N and pyrrolic N sites promote the reduction of NO_3_^−^ to generate the *NO intermediate, while graphitic N sites enhance CO_2_ adsorption and activation. By tuning the annealing temperature to optimize the N species ratio (pyridinic N: pyrrolic N: graphitic N ≈ 3:2:1), a balanced activity between NO_3_^−^RR and CO_2_RR was established, preventing excessive dominance of either reaction. The *NO and *CO intermediates then couple to form the key *OCNO intermediate, which subsequently undergoes a second C–N coupling and hydrogenation to produce urea. Meanwhile, Hu et al. [69] developed fluorine‐rich surface carbon nanotubes (F–CNT–300) via XeF_2_ treatment combined with annealing at 300°C, with C–F_2_ groups as the primary active sites on the surface (accounting for 93.9 at %). The high electronegativity of F induces charge inhomogeneity on the carbon matrix surface, significantly enhancing the co‐adsorption capability of NO_3_^−^ and CO_2_ (DFT calculations confirm that the formation of *NH and *CO intermediates is more favorable on the F–CNT surface). The interlayer spacing is expanded to 0.36–0.62 nm due to F doping, facilitating reactant mass transfer. At −0.65 V versus RHE, it achieves a UYR of 6.36 mmol h^−1^ g_cat_^−1^ and a FE of 18.0%, with performance 3.8 times that of undoped CNT, and both yield and selectivity remain stable after 8 cycles.

In summary, different types of active sites achieve differentiated division of labor and enhanced coupling in the synergistic NO_3_^−^RR and CO_2_RR reactions through electronic structure regulation and spatial configuration design. Single‐atom sites emphasize high atom utilization and precisely defined coordination environments; dual‐atom and multi‐atom sites optimize intermediate adsorption and reaction pathways via synergistic effects; alloys and heterostructures strengthen C–N coupling through interfacial electron transfer and strain effects; while MOF/COF and metal‐free systems exhibit unique advantages in confined microenvironments and multi‐site synergy. To intuitively compare the impact of different active site engineering strategies on catalytic performance, Figure 4g summarizes the distribution relationships of stability, UYR, and FE for the catalysts discussed in this section. It can be observed that MOF/COF and multi‐site synergistic systems exhibit more prominent overall performance, while single‐atom and certain heterostructure systems possess specific advantages in terms of stability or yield rate. However, high‐performance catalysts in the synergistic region of high stability and high yield rate still require further exploration. This further indicates that future catalyst design should shift from single‐performance optimization toward multi‐dimensional synergistic regulation of performance, in order to achieve the unification of high selectivity, high activity, and long‐term stability.

Despite these advances, each type of active site still faces specific limitations. SACs offer maximum atom utilization and well‐defined coordination environments, but a single atomic site can hardly satisfy the distinct adsorption requirements of NO_3_^−^RR and CO_2_RR simultaneously, and their high surface energy often leads to aggregation under operating conditions [34, 35]. DACs and multi‐atom sites partially overcome this limitation through site cooperation, yet the precise control of inter‐site distance and the scalable synthesis of uniform dual‐atom structures remain challenging [36, 48, 49]. Alloy and heterostructure catalysts provide effective interfacial electronic modulation, but their long‐term structural stability under harsh electrocatalytic conditions, particularly against surface reconstruction, leaching, and strain relaxation, still requires careful evaluation [37, 38, 39]. MOF/COF‐based catalysts benefit from confined microenvironments and tunable pore structures, but their intrinsically low electrical conductivity and limited stability in aqueous electrolytes constrain their practical application [40, 41, 42]. Metal‐free catalysts are cost‐effective and stable, but their Faradaic efficiency and urea yield rate are generally lower than those of metal‐based systems, and the precise identification of active sites remains difficult [43, 44]. Therefore, the rational design of urea electrosynthesis catalysts should not simply pursue a single type of active site, but rather integrate the complementary advantages of different site architectures to achieve simultaneous optimization of activity, selectivity, and stability.

#### 2.1.2 Support and Interface Optimization

Supports constitute an essential component of electrocatalysts for urea synthesis. They not only enhance the dispersion of active sites through high specific surface areas and porous structures, and improve electrical conductivity to optimize electron transfer efficiency, but also enable precise regulation of the electronic states of active sites via interfacial interactions with the active species. Moreover, certain supports can directly participate in the reaction through their own functional groups or structural features, such as assisting intermediate adsorption and constructing proton‐confined microenvironments, thereby synergistically promoting both CO_2_RR and NO_3_^−^RR as well as enhancing C–N coupling efficiency [70, 71, 72, 73].

##### 2.1.2.1 Carbon‐Based Supports

Carbon‐based supports have become the mainstream supports for electrocatalytic urea synthesis due to their combination of high electrical conductivity, large specific surface area, and favorable chemical stability. Zhang et al. [74] designed carbonized bacterial cellulose (CBC) prepared via high‐temperature carbonization of a bacterial cellulose aerogel, which inherits its 3D continuously interconnected fibrous network structure with a specific surface area of 162.4 m^2^ g^−1^ (Figure 5a,b). The abundant oxygen‐containing functional groups on the surface can form stable coordination bonds with Pd and Cu ions, which enables uniform anchoring of PdCu alloy nanoparticles and effectively suppresses particle agglomeration (Figure 5c,d). Meanwhile, the interfacial interaction between CBC and the PdCu alloy induces electron transfer between Pd and Cu (Bader charge analysis confirms a transfer amount of 1.65 e^−^), upshifting their d‐band centers and enhancing the adsorption and activation of CO_2_ and NO_3_^−^. This PdCu/CBC catalyst exhibits excellent performance in electrocatalytic urea synthesis, achieving a UYR of 763.8 ± 42.8 µg h^−1^ mg_cat_^−1^ at −0.50 V versus RHE and a maximum FE of 69.1 ± 3.8% at −0.40 V, with no significant performance decay after 10 h of continuous operation and 8 cycles, significantly outperforming single‐metal components (Pd/CBC, Cu/CBC) and conventional carbon black‐supported catalysts (PdCu/CB) (Figure 5e). Furthermore, Chen et al. [75] constructed interlayer iron atomic clusters (Fe_acs_) in expanded 2H‐graphitic carbon materials via a carbon defect confinement strategy, which not only provided stable anchoring sites for the Fe_acs_ clusters through strong Fe–C coordination but also created spatially matched nanoreactors. The carbon defects themselves can serve as auxiliary active sites, activating H_2_O to generate *H with a low energy barrier of 0.16 eV. Meanwhile, the strong interaction between Fe_acs_ and carbon defects optimizes the electronic structure, enhancing the adsorption energies of Fe_acs_ toward CO_2_, NO_3_^−^, and the *CO intermediate, significantly suppressing the *CO desorption side reaction, and reducing the energy barrier for dehydration of NH_2_OH to *NH_2_ to 0.19 eV. The synergy between the two sites accelerates C–N coupling. This system achieves a FE for urea of 39.80% and a normalized yield rate of 3643.65 mM h^−1^ g_Fe_^−1^, which are 7.98 and 9.88 times higher than those of Fe particles without interlayer structure, respectively. Moreover, after 58 h of continuous testing, the FE only decreases from 39.8% to 36.9%, demonstrating excellent stability. For the F–CNT–300 catalyst described in the preceding section on metal‐free catalysts, the carbon nanotubes construct efficient electron transport pathways by virtue of their one‐dimensional tubular structure, while the wall defects can synergize with metal active sites to adsorb *CO and *NH_2_ intermediates, further expanding the functional tunability of carbon‐based supports.

**FIGURE 5:**
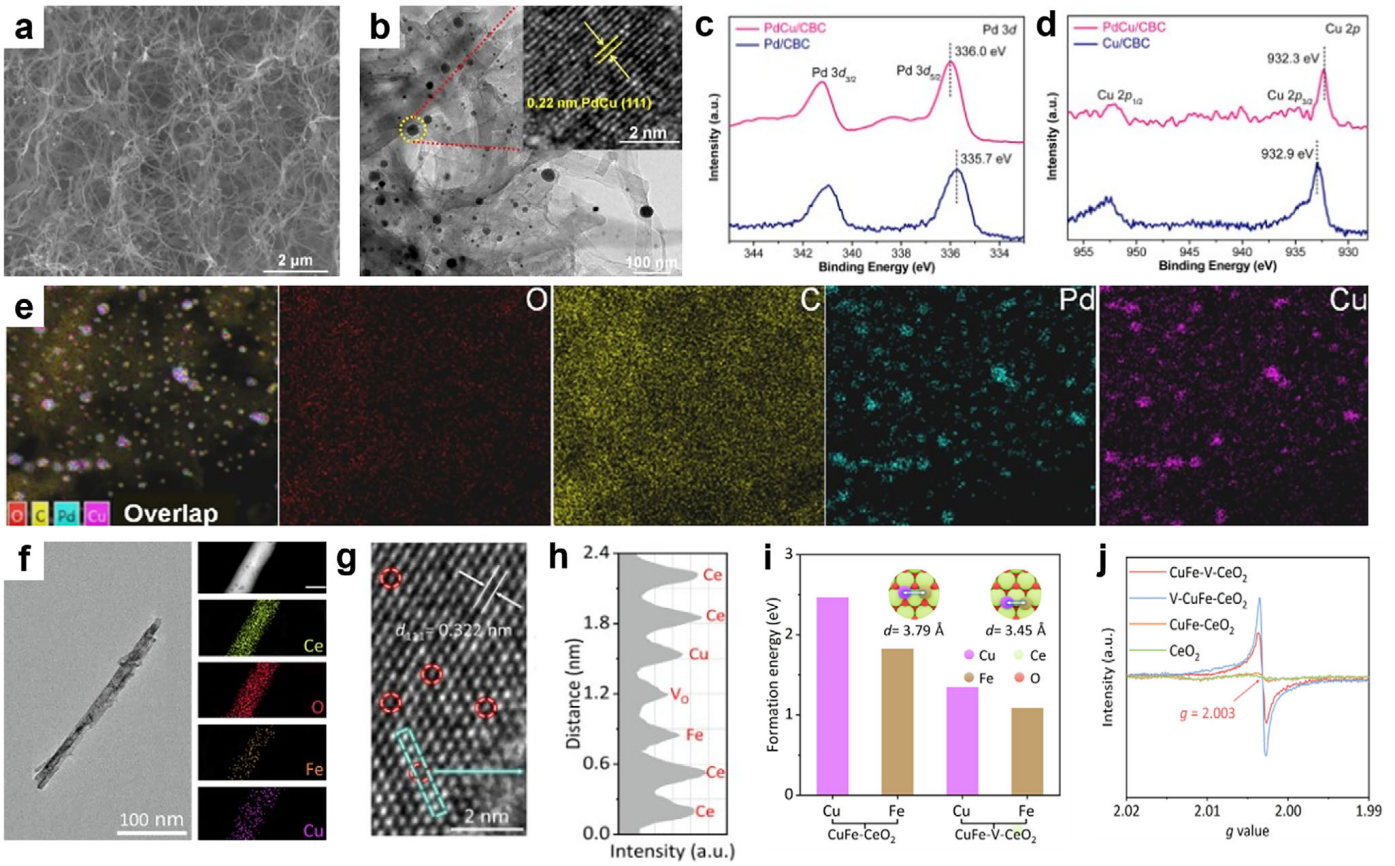
a) SEM and b) TEM images of the PdCu/CBC. c) Pd 3d XPS spectra of the Pd/CBC and PdCu/CBC. d) Cu 2p XPS spectra of the Cu/CBC and PdCu/CBC. e) Corresponding elemental mapping images of thePdCu/CBC. Reproduced with permission [74]. Copyright 2023, Royal Society of Chemistry. f) TEM and element mapping of CuFe‐V‐CeO_2_. g) HRTEM of CuFe‐V‐CeO_2_. h) Line‐scanning intensity profile on oxygen vacancy site in image (g). i) Formation energy of Cu and Fe on CuFe‐V‐CeO_2_ and CuFe‐V‐CeO_2_. j) EPR patterns of CeO_2_, CuFe‐CeO_2_, V‐CuFe‐CeO_2_, and CuFe‐V‐CeO_2_. Reproduced with permission [76]. Copyright 2025, Elsevier.

##### 2.1.2.2 Oxide Supports

Oxide supports have become important supports for electrocatalytic urea synthesis by virtue of their abundant oxygen vacancies (OVs) and tunable metal–support interactions. Their oxygen vacancies can enhance the adsorption and activation of NO_3_^−^, while simultaneously enabling precise regulation of the electronic states of active sites through interfacial electron transfer, thereby optimizing intermediate adsorption strength and reaction pathways [77, 78, 79]. Liu et al. [76] introduced oxygen vacancies into the CeO_2_ framework via a Joule heating method, which served as anchoring sites for Cu and Fe atoms to maximize atom utilization and formed active sites for urea synthesis (Figure 5f,g). The as‐prepared CuFe–V–CeO_2_ electrocatalyst features Cu–Fe interatomic distances that are well‐suited for C–N coupling (Figure 5h). The oxygen vacancy defects further enhance NO_3_^−^ adsorption, while interfacial electron transfer from Cu→Ce and Fe→Ce upregulates the electron density at Ce sites, promoting the generation of active hydrogen (H*) (Figure 5i,j). Meanwhile, the Fe sites strengthen CO_2_ adsorption and stabilization. The synergy among these three effects reduces the energy barrier of the rate‐limiting step for C–N coupling to 0.76 eV, achieving a UYR of 3553 mg h^−1^ g_cat_^−1^ at −1.5 V versus RHE, with no significant performance decay after 10 consecutive cycles. Similarly, Zhang et al. [80] designed a FeNC–Ce composite catalyst by dispersing FeNC onto an oxygen‐vacancy‐rich CeO_2_ support, constructing a dual‐active‐center synergistic system. The FeNC sites dominated NO_3_^−^ reduction, while the oxygen vacancies of CeO_2_ reinforced CO_2_ adsorption and activation, regulating the HER process from spontaneous to non‐spontaneous and suppressing competitive side reactions. This catalyst achieves an exceptionally high UYR of 20969.2 µg mg^−1^ h^−1^ and a FE of 89.3%, with no significant performance decay after 8 h of continuous electrolysis, demonstrating excellent activity and stability. In addition, Luo et al. [81] demonstrated that the residual lattice oxygen in oxide‐derived copper nanosheets (OL–Cu) can effectively modulate electron distribution, activating adjacent copper atoms and generating electron‐deficient copper sites (Cu^δ+^), which enhance CO_2_ adsorption capacity while stabilizing the *CO intermediate and directionally inducing NO_3_^−^ adsorption onto the adjacent Cu^δ+^ sites. This mechanism shortens the C–N coupling pathway, achieving a UYR of 298.67 mmol h^−1^ g^−1^ at −0.7 V versus RHE and an average FE of 31.71% at a high current density of approximately 95 mA cm^−2^. This study highlights the critical role of lattice oxygen in promoting adjacent co‐adsorption and enhancing C–N coupling selectivity.

In summary, support and interface engineering play a decisive role in determining the dispersion, electronic states, and local reaction microenvironment of active sites. Carbon‐based supports offer high electrical conductivity and large surface area, but their stability under prolonged operation, especially against carbon corrosion and surface oxidation, remains a concern. Oxide supports, particularly those with abundant oxygen vacancies, can enhance NO_3_^−^ adsorption and CO_2_ activation while modulating the electronic states of supported metals; however, they are susceptible to reduction and reconstruction under cathodic potentials, which may alter the active‐site configuration during operation. A key future direction is therefore to design supports that not only stabilize the active sites but also actively participate in the reaction through interfacial electronic coupling, proton confinement, or intermediate stabilization, thereby achieving synergistic optimization of both NO_3_^−^RR and CO_2_RR [70, 71, 72, 73, 74, 79].

### 2.2 Reaction Mechanism and Key Intermediate Analysis

The electrocatalytic synthesis of urea from NO_3_^−^ and CO_2_ is a complex heterogeneous catalytic process involving multi‐step proton‐coupled electron transfer (PCET) and targeted C–N bond coupling. The core mechanistic debate centers on the evolutionary pathways of key N/C‐containing intermediates and the modes of C–N coupling. The deep integration of in situ characterization techniques (in situ ATR‐SEIRAS, DEMS, XAS) with density functional theory (DFT) calculations provides essential support for dynamically tracking active site reconstruction, intermediate adsorption evolution, and quantifying reaction energy barriers [82, 83].

#### 2.2.1 Controversies and Consensus on Core Reaction Pathways

The currently accepted fundamental reaction framework is as follows: NO_3_^−^ undergoes multi‐step PCET to progressively generate N‐containing intermediates such as *NO, *NH_2_, and *NH_2_OH, while CO_2_ is simultaneously reduced via PCET to *CO. The two types of intermediates then undergo C–N coupling in the adjacent regions of active sites, followed by subsequent protonation and dehydration steps to ultimately form urea [84]. However, regulated by the type of active centers, coordination environments, and reaction conditions, the specific intermediate species and coupling pathways exhibit significant variations, giving rise to multiple mainstream reaction mechanisms.

##### 2.2.1.1 *CO and *NH_2_ Coupling Mechanism

This mechanism is currently the most thoroughly studied and broadly applicable core pathway, and has been validated in Cu‐based, Fe‐based catalysts, as well as single‐atom/dual‐atom catalytic systems [51, 85, 86]. Its core feature lies in the requirement that NO_3_^−^ and CO_2_ undergo deep reduction to generate the key intermediates *NH_2_ and *CO, respectively, which then achieve C–N coupling through spatial proximity effects. Specifically, NO_3_^−^ reduction via multi‐step PCET follows the main sequence of *NO_3_ → *NO_2_ → *NO → *NHO → *NH_2_. Based on the conversion pathway from *NHO to *NH_2_, this process can be further subdivided into three pathways: Pathway I (*NHO → *NHOH → *NH →*NH_2_) [69, 86, 87, 88, 89, 90], Pathway II (*NHO → *NHOH → *NH_2_OH →*NH_2_) [91], and Pathway III (*NHO → *N → *NH → *NH_2_) [92], among which Pathway I is the most common due to its thermodynamic advantages. Recent in situ DEMS studies have directly detected key N‐containing intermediates such as *NHOH, *NH, and *NH_2_ during NO_3_^−^RR, providing experimental evidence for these hydrogenation pathways [93, 94]. Meanwhile, CO_2_ generates carbon‐containing intermediates via the reduction route of *CO_2_ → *COOH → *CO, with the energy barrier for the *COOH → *CO step typically ranging from 0.4 to 0.6 eV [95]. The two types of intermediates undergo targeted coupling in the adjacent regions of catalytic sites to form *CONH_2_, which is then converted to urea through subsequent protonation steps. The rate‐determining step (RDS) of this mechanism is catalyst‐dependent and may be *CO formation, the *NHO → *NHOH transformation, or the second C–N coupling. In situ FTIR can detect characteristic vibrational peaks at ∼2091 cm^−1^ (*CO) and ∼1137 cm^−1^ (*NH_2_), providing direct evidence for mechanistic validation (Figure 6) [90].

**FIGURE 6:**
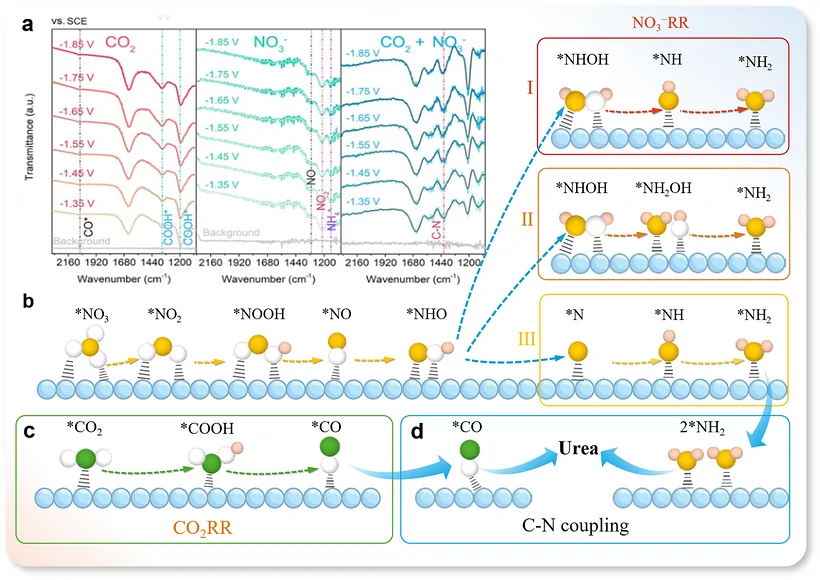
In situ ATR‐FTIR spectra of a) NO_3_^−^, CO_2_, and a mixture of NO_3_^−^. Reproduced with permission [90]. Copyright 2022, American Chemical Society. Mechanism of b) NO_3_^−^RR, c) CO_2_RR, and d) C─N coupling of *CO and *NH_2_ from CO_2_RR and NO_3_^−^RR.

##### 2.2.1.2 *CO and *NO Coupling Mechanism

Different from the *CO‐*NH_2_ coupling mechanism, the C–N coupling in this pathway occurs at an early stage of NO_3_^−^ reduction, without the need for deep reduction to *NH_2_, effectively reducing the reaction energy consumption. The reaction logic is as follows: NO_3_^−^ undergoes rapid reduction to generate the *NO intermediate, while CO_2_ is simultaneously reduced to *CO. The two species preferentially couple to form the key intermediate *OCNO, which subsequently undergoes multi‐step structural rearrangement and protonation/dehydration processes to sequentially generate *ONCONOH, *NCON, *NHCONH_2_, and other intermediates, ultimately converting to urea [78, 96, 97, 98, 99]. Based on the differences in the subsequent hydrogenation sequence and hydroxyl removal pathways of *OCNO, this mechanism can be further subdivided into three pathways: Pathway IV (*ONCONOH → *HONCONOH → *NCONOH → *NHCONH_2_) [96], Pathway V (*ONCONOH → *ONCON → *ONCONH → *NHCONH_2_) [78], and Pathway VI (*ONCONOH → *ONCON → *HONCON → *NHCONH_2_) [97]. The core advantage of this mechanism lies in its lower coupling energy barrier; however, direct in situ characterization evidence for the intermediates involved in the subsequent transformation of *OCNO is lacking, and the pathway mainly relies on DFT calculations and product origin verification (Figure 7).

**FIGURE 7:**
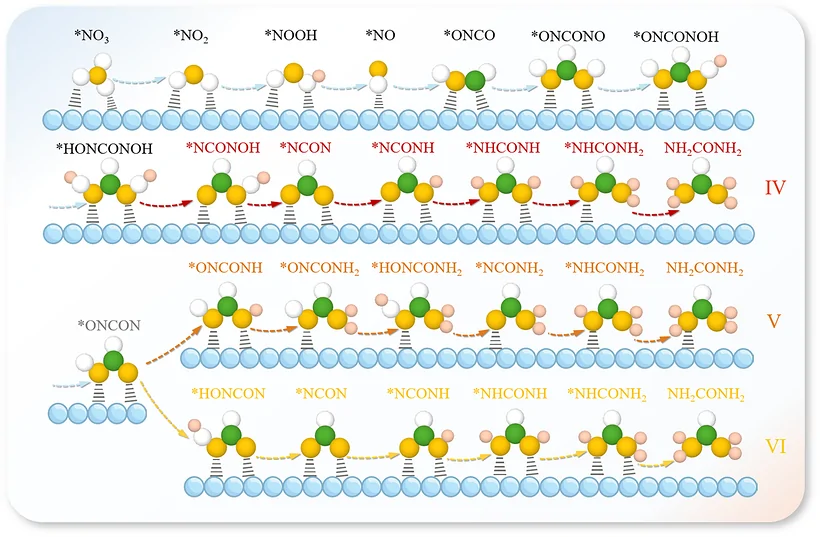
Mechanism of C─N coupling of *CO and *NO from CO_2_RR and NO_3_^−^RR.

##### 2.2.1.3 *CO Coupling with Other N‐Containing Intermediates

This mechanism breaks through the limitation of relying solely on *NH_2_ and *NO as N‐containing coupling intermediates, expanding the diversified pathways of C–N coupling. Its core lies in the fact that intermediates generated during NO_3_^−^ reduction, such as *NO_2_, *NOOH, *NOH, and *NH, can all undergo efficient coupling with *CO, circumventing the high energy consumption associated with deep reduction in conventional pathways [99, 100, 101, 102]. The clearly identified specific pathways to date include: VII) *CO coupling with *NO_2_: generating the *CONO_2_ intermediate, which undergoes protonation via *NO_2_CONH_2_ to form urea [100]; VIII) *CO coupling with *NOOH: forming the *CONOOH intermediate, which is reduced in three steps to *CON, followed by a second coupling with another *NOOH molecule to complete urea synthesis [101]; IX) *CO coupling with *NOH: DFT calculations confirm that the coupling energy barrier of this pathway is lower than that of other N‐containing intermediates, generating *NOHCO, which is protonated to *NCO, followed by a second C–N coupling with *NOH [102]; X) *CO coupling with *NH: thermodynamically spontaneous formation of the *NHCO intermediate, which then couples with *NO to generate *NHCONO, ultimately forming urea via PCET steps [103]. The key to such mechanisms lies in the ability of catalytic sites to precisely regulate the evolution of NO_3_^−^ reduction intermediates, preventing excessive reduction to byproducts such as NH_3_ (Figure 8).

**FIGURE 8:**
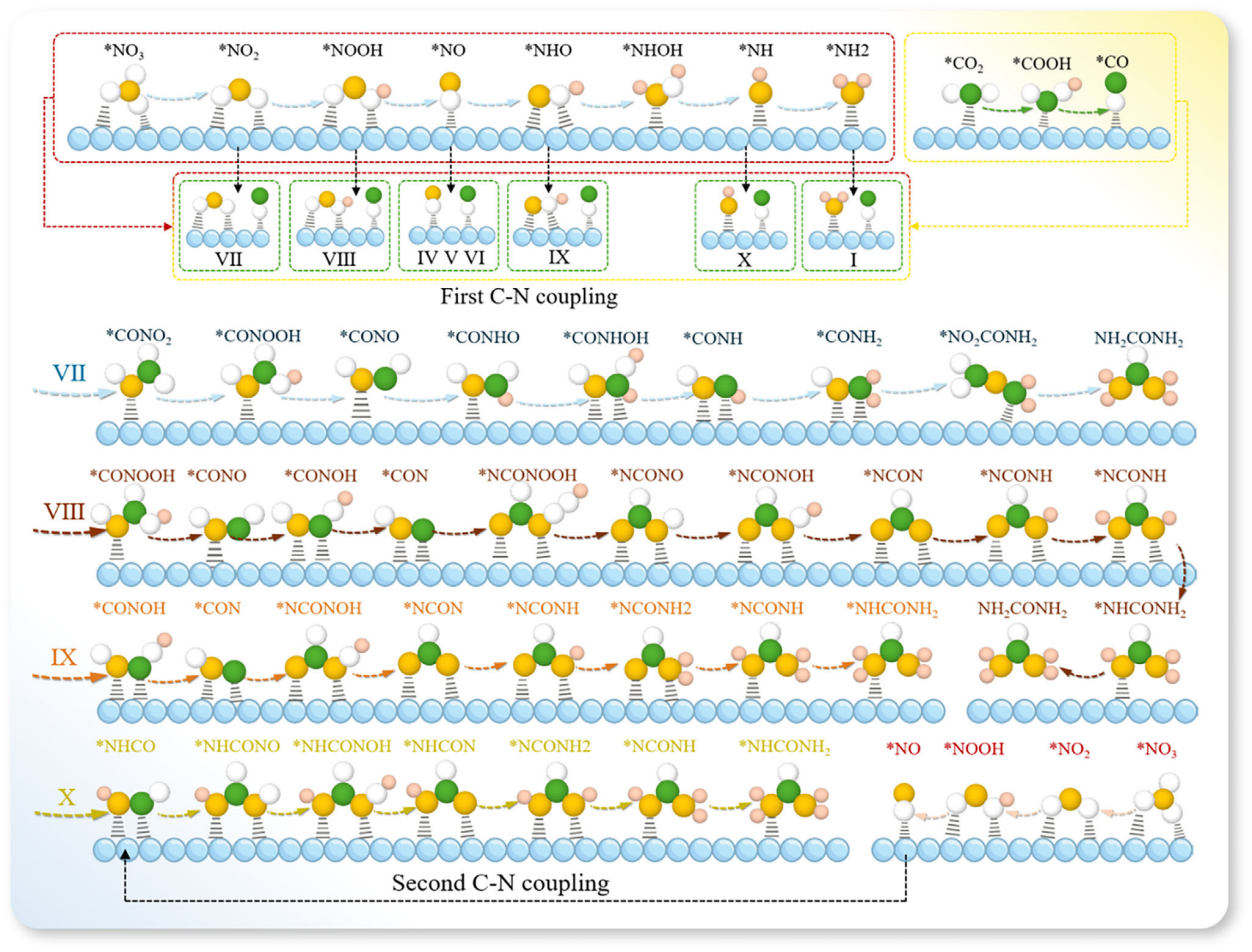
Mechanism of C─N coupling of *CO and other N‐containing intermediates from CO_2_RR and NO_3_^−^RR.

##### 2.2.1.4 Direct Coupling Mechanism of CO_2_ with N‐Containing Intermediates

This mechanism breaks the conventional understanding that CO_2_ must first be reduced to *CO before participating in coupling. Instead, CO_2_ directly serves as the carbon source to undergo C–N coupling with N‐containing intermediates generated from NO_3_^−^ reduction, significantly simplifying the reaction steps and enhancing CO_2_ utilization efficiency [104]. The reported specific pathways mainly include three types: XI) Direct coupling of CO_2_ with *NO: generating the *CO_2_NO_2_ intermediate, which undergoes protonation via *CO_2_NH → *COOHNH_2_ to form urea [83, 105]; XII) Coupling of CO_2_ with NO_2_^−^: NO_3_^−^ is first reduced to NO_2_^−^, which then combines with CO_2_ to form *CO_2_NO_2_, subsequently converted to urea via *CO_2_NH_2_, with thermodynamic calculations confirming that all steps are exothermic [106]; XIII) Coupling of CO_2_ with *NH: directly generating the *NHCO_2_ intermediate, which is reduced to *NHCO and then undergoes a second coupling with *NH_2_ to form urea [107]. Such mechanisms have currently only been verified in specific catalytic systems, such as In(OH)_3_ and Vitamin B_12_‐functionalized carbon nanotubes. The core challenge lies in how to enhance CO_2_ activation and the targeted generation of N‐containing intermediates, while preventing CO_2_ from being reduced to byproducts such as CO or HCOOH (Figure 9) [106, 108].

**FIGURE 9:**
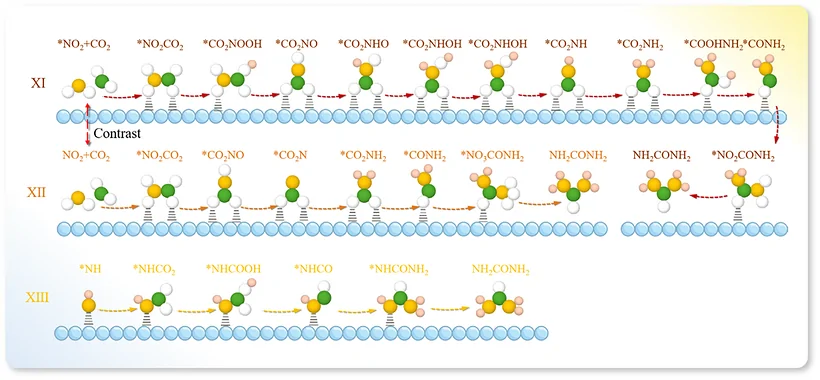
Mechanism of C─N coupling of CO_2_ and N‐containing intermediates from NO_3_^−^RR.

Taken together, the mechanistic understanding of C–N coupling in electrocatalytic urea synthesis from NO_3_^−^ and CO_2_ is gradually evolving from predominantly theory‐driven mechanistic proposals toward increasingly evidence‐based reaction networks. Nevertheless, the level of experimental validation remains highly uneven among the pathways summarized in Figures 7, 8, 9. While these mechanistic frameworks provide valuable insights into possible C–N coupling routes, many transient intermediates and elementary reaction steps are still supported primarily by density functional theory (DFT) calculations combined with indirect spectroscopic observations, rather than direct experimental verification. Recent advances in operando and isotope‐resolved characterization techniques have substantially strengthened mechanistic investigations of electrocatalytic urea synthesis. For nitrate reduction, operando synchrotron‐radiation Fourier‐transform infrared spectroscopy (SR‐FTIR), online differential electrochemical mass spectrometry (DEMS), and isotope‐labelled DEMS have enabled the direct identification of key nitrogen‐containing intermediates, including *NO, *NHO, *NHOH, and *NH_2_OH. Furthermore, potential‐dependent DEMS measurements have revealed the dynamic formation of *NOOH and its strong dependence on active‐hydrogen (*H) availability, providing direct experimental evidence for catalyst‐dependent hydrogenation pathways of nitrogen‐containing intermediates [109, 110]. More importantly, for electrocatalytic urea synthesis via CO_2_/NO_3_^−^ co‐reduction, isotope‐labelled DEMS has directly detected CO^+^, NH_2_^+^, CONH_2_^+^, and CO^15^NH_2_^+^ fragments, while complementary operando SR‐FTIR and in situ Raman spectroscopy have independently identified CO‐, NH_2_‐, and C–N‐related vibrational signatures, providing compelling experimental evidence for the involvement of key C–N coupling intermediates [111]. These advances demonstrate that mechanistic understanding is increasingly supported by direct experimental observations rather than solely by theoretical predictions. Nevertheless, the direct identification of several short‐lived reaction intermediates and complete elementary reaction sequences remains challenging. Future mechanistic investigations should therefore integrate isotope‐resolved mass spectrometry, operando vibrational spectroscopy, and theoretical calculations to establish more rigorous structure–intermediate–activity relationships and to further validate the proposed reaction pathways.

#### 2.2.2 Proton Regulation and Side‐Reaction Suppression

The supply rate and spatial distribution of protons are core factors in regulating the synergy among NO_3_^−^RR, CO_2_RR, and C–N coupling. Insufficient proton supply leads to incomplete hydrogenation of intermediates, while excessive supply tends to trigger competitive side reactions. Meanwhile, the presence of side reactions such as HER, over‐reduction of NO_3_^−^ to NH_3_, and reduction of CO_2_ to CO/HCOOH can divert reactants and active sites, directly diminishing the selectivity and Faradaic efficiency of urea synthesis.

Proton regulation and side‐reaction suppression are core strategies for optimizing the performance of NO_3_^−^–CO_2_ electrocatalytic urea synthesis. Proton regulation centers on electrolyte composition optimization and catalyst microenvironment design. HCO_3_^−^ in KHCO_3_ can maintain pH stability and precisely supply protons via the HCO_3_^−^ → *COOH pathway [112]. The 2–5 nm porous structures of MOFs/COFs can construct proton‐confined microenvironments to slow H diffusion, suppress HER, and ensure the proton demand for coupling [54, 64]. Ionic liquid additives can increase CO_2_ solubility by a factor of three and regulate proton transport, thereby enhancing the selectivity of urea electrosynthesis [113]. Side‐reaction suppression is primarily achieved through modulating the electronic states of active sites and the adsorption strength of intermediates. HER suppression can be realized by increasing the hydrogen adsorption energy or constructing hydrophobic environments [114, 115]. Suppression of NO_3_^−^ reduction byproducts require enhanced adsorption of N‐containing intermediates or shortened spatial distances to C‐containing intermediates. Suppression of CO_2_ reduction byproducts can be achieved by tuning the d‐band center of active sites to strengthen *CO adsorption [32, 33].

### 2.3 Performance Optimization and Engineering Exploration

#### 2.3.1 Reaction Condition Optimization

Reaction condition optimization is key to enhancing the activity and selectivity of NO_3_^−^–CO_2_ electrocatalytic urea synthesis, and mainly focuses on two aspects: electrolyte optimization and reactor design. In terms of electrolyte, the KHCO_3_/NaHCO_3_ system is predominantly employed, which not only provides a stable proton‐buffering environment for the reaction but also enhances CO_2_ solubility [112, 116, 117]. The NO_3_^−^ concentration also needs to be controlled within a certain range, as excessively low concentrations lead to insufficient supply of N‐containing intermediates, while excessively high concentrations promote the formation of byproducts such as NH_3_ [118, 119]. The addition of a certain concentration of KCl solution to the electrolyte can achieve multiple optimizations: on one hand, K^+^ and Cl^−^ enhance the ionic mobility of the electrolyte, thereby improving conductivity; on the other hand, Cl^−^ adsorbed on the catalyst surface suppresses HER, thereby enhancing the selectivity toward urea [118, 120, 121]. In terms of reactor design, the conventional H‐cell suffers from low mass transfer efficiency that limits the reaction rate, making the dual‐chamber membrane electrode assembly (DCMEA) and flow cell reactor more favorable alternatives (Figure 10a–c) [122]. For example, the DCMEA employs an anion exchange membrane to separate the anode and cathode, facilitating efficient contact between CO_2_ and NO_x_^−^ with the catalyst on the cathode side, enabling the Ru_1_/CuO_x_ system to achieve a UYR of 106.9 mmol h^−1^ g_cat_^−1^, which is six times higher than that in the H‐cell (Figure 10d) [17]. The flow cell reactor utilizes a gas diffusion electrode (GDE), allowing CO_2_ to directly contact the catalyst in the gaseous state, enhancing solubility by more than tenfold. The Cu/Cu_2_O system achieves a UYR of 883.6 µg h^−1^ mg_cat_^−1^ in this reactor, representing a 40% improvement over the H‐cell (Figure 10e) [123].

**FIGURE 10:**
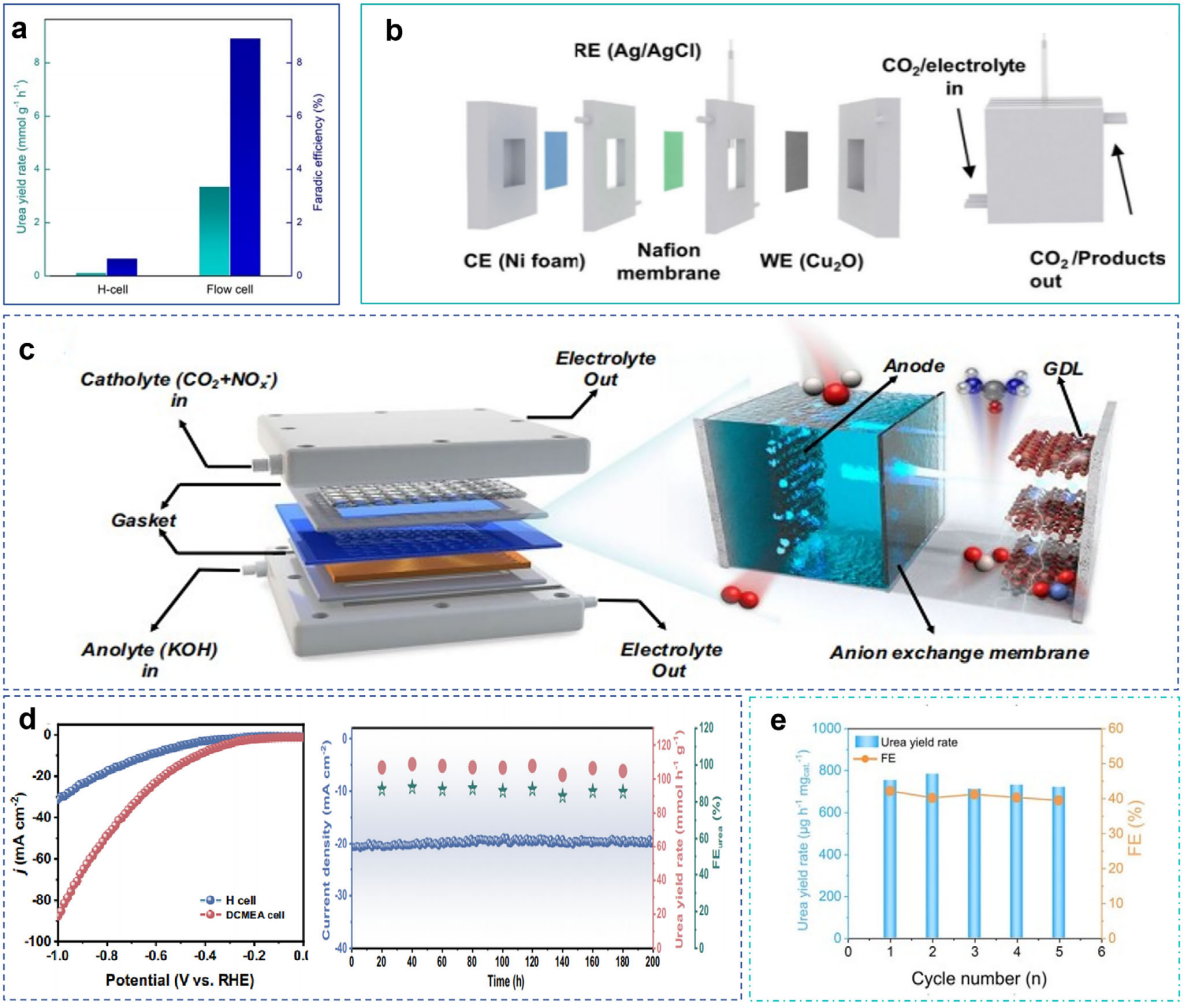
a) Comparison of electrocatalytic performance in a H‐cell and a flow cell. Reproduced with permission [122]. Copyright 2021, Wiley‐VCH. b) Schematic diagram of a three‐electrode flow cell. Reproduced with permission [123]. Copyright 2025, Springer Nature. c) Schematic of the DCMEA cell. Reproduced with permission [17]. Copyright 2025, Springer Nature. d) LSV curves of Ru_1_/CuO_x_ recorded in different cells, chronoamperometry tests of Ru_1_/CuO_x_ performed at −0.6 V for 200 h, as well as the corresponding urea yield rates and Faradaic efficiencies for urea at different reaction times. e) Consecutive urea synthesis stability test at −0.3 V versus RHE over h‐Cu/Cu_2_O MPs in a three‐electrode flow cell.

#### 2.3.2 Dynamic Regulation Techniques

Dynamic regulation techniques, through real‐time dynamic modulation of external fields such as potential and magnetic fields, precisely match the generation, stabilization, and coupling requirements of intermediates in the multi‐step NO_3_^−^–CO_2_ electrocatalytic urea synthesis, effectively addressing the kinetic mismatch issues inherent to static catalysis [124, 125, 126, 127, 128, 129, 130, 131, 132]. Pulsed potential regulation is centered on the periodic switching between high and low potentials. The high potential region could directionally promote NO_3_^−^ reduction, stabilizing key N‐containing intermediates and preventing their over‐reduction to byproducts. The low potential region lowers the energy barriers for C–N coupling and protonation, facilitating the targeted reaction between N‐containing intermediates and *CO. The Au@cpCu_7_CF system achieves a FE as high as 55.53% under pulsed potential, with an optimal pulse period of 2–4 s [133]. Magnetic field–electrocatalysis coupling optimizes the reaction based on the spin‐catalysis mechanism. Using the dilute magnetic oxide Cu–In_2_O_3_ as the catalyst, the magnetic field can orient the spin of Cu sites, inducing spin polarization of adsorbed CO_2_ and enabling antiparallel spin coupling with *NH_2_ via hydrogen bonding, which promotes N–H bond cleavage and O–H bond formation during hydrogen transfer, while simultaneously eliminating the limiting potential for urea formation [134]. This system achieves a UYR of 34.5 mmol h^−1^ g^−1^, which is 2.2 times higher than that without magnetic field regulation, with the FE increasing from 14.9% to 24.3%. In a flow cell, an even higher UYR of 83.2 mmol h^−1^ g^−1^ is achieved, with stable performance over 10 h of continuous electrolysis.

#### 2.3.3 Performance Benchmarking and Data Comparison

Significant progress has been achieved in the core performance metrics (FE, yield rate, and stability) of the NO_3_^−^–CO_2_ system, and Table 1 summarizes the representative achievements in recent years.

**TABLE 1:** Representative performance metrics of NO_3_^−^–CO_2_ electrocatalytic urea synthesis.

Catalyst	Electrolyte	Potential (vs. RHE)	Current density	FE	Original UYR (Reactor)	Unified UYR	Stability	Refs
Ru_1_/CuO_x_ (SACs)	0.1 M NO_x_^−^ + 0.1 M KHCO_3_ (CO_2_ saturation)	−0.6 V	≈ −20 mA cm^−2^	86.7%	106.9 mmol·h^−1^·g_cat_^−1^ (DCMEA)	106.9 mmol h^−1^·g_cat_^−1^	200 h	[17]
Cu‐N_3_ (SACs)	0.1 M KHCO_3_ + 0.1 M KNO_3_ (CO_2_ saturation)	−0.9 V	≈ −28 mA mA cm^−2^	55.4%	19597.65 µg·h^−1^·mg_Cu_^−1^ (H‐cell)	7.93 mmol h^−1^·g_cat_^−1^	10 h	[45]
Ga/Y‐CNP (DACs)	0.1 M KHCO_3_ + 0.05 M KNO_3_ (CO_2_ saturation)	−1.4 V	≈ −5 mA cm^−2^	22.1%	41.9 mmol·h^−1^·g_cat_^−1^ (H‐cell)	41.9 mmol h^−1^·g_cat_^−1^	120 h	[50]
SrCo_0.39_Ru_0.61_O_3‐δ_ (DACs)	0.1 M KNO_3_ (CO_2_ saturation)	−0.7 V	≈ −25 mA cm^−2^	34.1%	1522 µg·h^−1^·mg_cat_^−1^ (H‐cell)	25.34 mmol h^−1^·g_cat_^−1^	360 h	[51]
PdCuCo (Polyatomic)	0.1 M KHCO_3_ + 0.1 M KNO_3_	−0.6 V	≈ −11 mA cm^−2^	81%	227 mmol·h^−1^·g_cat_^−1^ (H‐cell)	227 mmol h^−1^·g_cat_^−1^	> 260 h	[52]
Sn/Cu_2_O (Heterogeneous)	0.05 M KNO_3_ + 0.1 M KHCO_3_ (CO_2_ saturation)	−0.4 V	≈ −20 mA cm^−2^	13.5%	32.35 µmol·h^−1^·cm^−2^ (GDE)	80.9 mmol h^−1^·g_cat_^−1^	10 h	[58]
C‐Cu‐A_4_ (Heterogeneous)	0.9 M KHCO_3_ + 0.1 M KNO_3_ (CO_2_ saturation)	−1.2 V	≈ −20 mA cm^−2^	45.4%	0.89 mol·h^−1^·g^−1^ (H‐cell)	890 mmol h^−1^·g_cat_^−1^	30 h	[53]
Cu_2_Sb (Alloy)	0.1 M KHCO_3_ + 0.1 M KNO_3_ (CO_2_ saturation)	−0.4 V	≈ −20 mA cm^−2^	64.9%	22.9 mmol·h^−1^·g_cat_^−1^ (H‐cell)	22.9 mmol h^−1^·g_cat_^−1^	200 h	[59]
PdAuCuIrCo (Alloy)	0.02 M KNO_3_ (CO_2_ saturation)	−0.9 V	≈ −5 mA cm^−2^	22.57%	52.43 mmol·h^−1^·g^−1^ (H‐cell)	52.43 mmol h^−1^·g_cat_^−1^	6 cycles	[60]
PCOF‐34‐Fe (COF)	0.1 M KHCO_3_ + 0.1 M KNO_3_ (CO_2_ saturation)	−0.5 V	≈ −12 mA cm^−2^	90%	135.6 mmol·h^−1^·g_cat_^−1^ (GDE)	135.6 mmol h^−1^·g_cat_^−1^	> 100 h	[64]
PcNi‐Fe‐O (MOF)	0.1 M KNO_3_ (CO_2_ saturation)	−0.6 V	≈ −10.1 mA cm^−2^	54.1%	2.1 g·h^−1^·g_cat_^−1^ (GDE)	34.97 mmol h^−1^·g_cat_^−1^	20 h	[54]
N‐C‐1000 (Metal‐free)	0.1 M KHCO_3_ + 10 mM KNO_3_ (CO_2_ saturation)	−1.5 V	≈ −15 mA cm^−2^	12%	610.6 mg·h^−1^·g^−1^ (H‐cell)	10.17 mmol h^−1^·g_cat_^−1^	6 cycles	[68]
F‐CNT‐300 (Metal‐free)	0.1 M KNO_3_ (CO_2_ saturation)	−0.65 V	≈ −0.5 mA cm^−2^	18.0%	6.36 mmol·h^−1^·g_cat_^−1^ (H‐cell)	6.36 mmol h^−1^·g_cat_^−1^	8 cycles	[69]
PdCu/CBC (Carbon‐based)	0.05 M KNO_3_ (CO_2_ saturation)	−0.5 V	≈ −5 mA cm^−2^	59.7%	0.764 mmol·h^−1^·g_cat_^−1^ (H‐cell)	12.72 mmol h^−1^·g_cat_^−1^	10 h	[74]
Fe_acs_ (Carbon‐based)	0.25 M KNO_3_ + 0.1 M KHCO_3_ (CO_2_ saturation)	−0.64 ∼ −1.04 V	≈ −2 mA cm^−2^	39.8%	3643 mmol·h^−1^·g_Fe_^−1^ (H‐cell)	37.17 mmol h^−1^·g_cat_^−1^	58 h	[75]
CuFe‐V‐CeO_2_ (Oxide carrier)	0.1 M KHCO_3_ + 50 mM KNO_3_ (CO_2_ saturation)	−1.5 V	≈ −20 mA cm^−2^	8.8%	3553 mg·h^−1^·g_cat_^−1^ (H‐cell)	59.16 mmol h^−1^·g_cat_^−1^	10 cycles	[76]
FeNC‐CeO_2_ (Oxide carrier)	1 M KNO_3_+0.1 M KHCO_3_	−0.5 V	≈ −20 mA cm^−2^	89.3%	349.1 mmol·h^−1^·g_cat_^−1^ (H‐cell)	349.13 mmol h^−1^·g_cat_^−1^	8 h	[80]
OL‐Cu (Oxide carrier)	0.9 M KHCO_3_ + 0.1 M KNO_3_ (CO_2_ saturation)	−0.7 V	≈ −95 mA cm^−2^	28.64%	298.67 mmol·h^−1^·g^−1^ (H‐cell)	298.67 mmol h^−1^·g_cat_^−1^	8 cycles	[81]
Cu/Cu_2_O (Reactor utilization)	0.5 M KHCO_3_ + 0.05 M KNO_3_ (CO_2_ saturation)	−0.3 V	≈ −20 mA cm^−2^	42.3%	632.1 µg·h^−1^·mg_cat_^−1^ (GDE)	10.52 mmol h^−1^·g_cat_^−1^	12 h	[123]
Au@cpCu_7_CF (Dynamic regulation)	8 mM KNO_3_+0.2 M K_2_SO_4_ (CO_2_ saturation)	Pulse (−0.6 V/−0.2 V)	≈ −0.3 mA cm^−2^	55.53%	125.70 mg·g^−1^·h^−1^ (H‐cell)	2.09 mmol h^−1^·g_cat_^−1^	11700 Periodic stability	[133]
Cu‐In_2_O_3_ (Dynamic regulation)	0.1 M KNO_3_ (CO_2_ saturation)	−0.5 V	≈ −10 mA cm^−2^	33.27%	3779.26 µg·h^−1^·mg_cat_^−1^ (H‐cell)	62.92 mmol h^−1^·g_cat_^−1^	15 cycles	[134]

Before comparing the performance metrics across different NO_3_^−^ + CO_2_ systems, it is necessary to clarify how the FE is calculated. For this system, the overall urea‐forming reaction involves 16 electrons per urea molecule:

(1) $$2N{O_{3}}^{-}+CO_{2}+18H^{+}+16e^{-}\to \mathrm{CO}{\left(NH_{2}\right)}_{2}+7H_{2}O$$

The FE is therefore calculated as:

(2) $$FE_{\mathrm{urea}}\left(\%\right)=\frac{16\times F\times C_{\mathrm{urea}}\times V}{M_{\mathrm{urea}}\times Q}\times 100\%$$

where F is the Faraday constant (96485 C mol^−1^), C_urea_ is the measured urea concentration, V is the electrolyte volume, M_urea_ is the molecular weight of urea (60.06 g mol^−1^), and Q is the total charge passed during electrolysis [78, 83]. It should be noted that the measured FE is strongly influenced by reactor configuration, electrolyte composition, nitrate concentration, CO_2_ supply mode, and quantification method, and thus cross‐study comparison should be made with caution [135, 136].

In recent years, the NO_3_^−^ + CO_2_ electrocatalytic urea synthesis system has achieved substantial breakthroughs in key metrics such as Faradaic efficiency, urea yield rate, and operational stability, yet the performance advantages exhibited by different catalyst systems are clearly differentiated. In terms of selectivity, PCOF‐34‐Fe, FeNC−CeO_2_, and Ru_1_/CuO_x_ achieved FE values of 90%, 89.3%, and 86.7%, respectively, reflecting the significant advantages of dual‐site synergistic construction, oxygen vacancy regulation, and confined pore microenvironment design in reconciling the kinetics of NO_3_^−^RR and CO_2_RR, promoting targeted coupling of key intermediates, and suppressing competitive reactions such as HER and ammonia formation. In terms of yield performance, systems such as PcNi−Fe−O, FeNC−CeO_2_, OL−Cu, PdCuCo, and PCOF‐34‐Fe exhibit strong urea production capability [52, 54, 64, 80, 81], indicating that multi‐active‐site division of labor, interfacial electronic structure modulation, and enhanced reactor mass transfer can effectively improve C−N coupling efficiency and accelerate the overall reaction process. It should be noted that, since different studies have adopted various normalization methods for yield rates, such as based on catalyst mass, mass of active metal components, geometric area, or specific reactor configurations, the data in the table are more suitable for revealing the performance hierarchy and design principles of different systems, rather than for simple horizontal ranking based on absolute values. From the perspective of stability, catalysts such as SrCo_0.39_Ru_0.61_O_3‐δ_, PdCuCo, Ru_1_/CuO_x_, Cu_2_Sb, and PCOF‐34‐Fe have achieved continuous or cyclic stable operation on the scale of over 100 h and even up to several hundred hours [17, 51, 52, 59, 64], indicating that the combination of high activity and long‐term structural stability is gradually transitioning from conceptual validation to reproducible realization. Further analysis in conjunction with reactor conditions reveals that, compared with the conventional H‐cell, enhanced mass transfer systems such as DCMEA and GDE offer greater advantages in improving reactant supply efficiency, reducing concentration polarization, and enhancing interfacial reaction rates. For example, Ru_1_/CuO_x_ achieves a UYR of 106.9 mmol h^−1^ g_cat_^−1^ in the DCMEA with stable operation over 200 h, while PCOF‐34‐Fe attains a UYR of 135.6 mmol h^−^^1^ g_cat_^−1^ in the GDE system. This indicates that reactor engineering is no longer merely an auxiliary factor for performance scale‐up, but rather a critical variable that influences the overall performance of the catalytic system. Overall, research on NO_3_^−^ + CO_2_ electrocatalytic urea synthesis is transitioning from the early pursuit of a single high FE or high yield rate toward a comprehensive development stage that emphasizes synergistic optimization of selectivity, yield, stability, and engineering compatibility. In the future, further breakthroughs of this system will still rely on the precise construction of active sites, accurate modulation of interfacial electronic states, and deep coupling between catalytic materials and reactor architectures.

## 3 Electrocatalytic Urea Synthesis System of N_2_ + CO_2_

The electrocatalytic synthesis of urea from N_2_ and CO_2_, with feedstocks derived from the infinitely renewable N_2_ in the atmosphere and industrially emitted CO_2_, can not only achieve green cycling of carbon and nitrogen resources but also circumvent the limitation of NO_3_^−^ feedstocks relying on fossil fuel‐based production, making it the core direction for the long‐term development of future sustainable urea synthesis technologies [137−143]. However, the practical application of this system still faces formidable core challenges. On one hand, the N≡N bond energy in the N_2_ molecule is as high as 941 kJ mol^−1^, far exceeding the N = O bond energy in NO_3_^−^ (204 kJ mol^−1^), rendering it highly chemically inert and difficult to be efficiently adsorbed and activated by catalysts under ambient temperature and pressure, leading to extremely high kinetic barriers for the N_2_ reduction reaction [22, 144, 145]. On the other hand, the N‐containing intermediates generated from N_2_ reduction (e.g., *N_2_, *NNH, *NH_2_) suffer from poor stability and are prone to protonation to form byproducts such as NH_3_, while the C‐containing intermediates generated from CO_2_ reduction (e.g., *CO, *COOH) have short lifetimes and are susceptible to desorption to form CO or methane. The generation rates, adsorption strengths, and spatial distributions of the two types of intermediates on the catalyst surface are difficult to precisely match, resulting in extremely low C−N coupling efficiency [12, 13, 100, 146−149]. These factors collectively result in the urea yield rate and Faradaic efficiency of the N_2_ + CO_2_ system being far lower than those of the NO_3_^−^ + CO_2_ system, constituting the key bottleneck that constrains the practical application of this technology [150−154].

### 3.1 Core of Catalyst Design: Synergy Between N_2_ Activation and C–N Coupling

The catalyst design for the N_2_ + CO_2_ electrocatalytic urea synthesis system must overcome the limitations of single functionality and achieve synergistic coordination among the three key steps of N_2_ activation, CO_2_ reduction, and C−N coupling. The core of its design lies in the precise regulation of the electronic states and coordination environments of active sites. On one hand, it is necessary to weaken the N≡N bond strength in the N_2_ molecule, reduce its activation energy barrier, and promote the adsorption and dissociation of N_2_. On the other hand, it is essential to optimize the adsorption strength and residence time of N‐containing and C‐containing intermediates on the catalyst surface, preventing excessive protonation or desorption loss of intermediates. Meanwhile, it is also necessary to regulate the spatial distribution and inter‐site distance of active sites to ensure the matching of generation rates of the two types of intermediates at the catalytic interface, thereby providing thermodynamic and kinetic advantages for efficient C−N coupling [20, 155−159].

#### 3.1.1 Noble Metal‐Based Catalysts

Noble metals (Ru, Pd, Pt, etc.), by virtue of their tunable d‐band centers and excellent surface adsorption and activation capabilities, have become classic research subjects for the N_2_ + CO_2_ electrocatalytic urea synthesis system. The core advantage of these catalysts lies in the fact that their d‐orbital electrons can effectively overlap with the π* antibonding orbitals of N_2_ molecules, weakening the N≡N bond strength, while simultaneously providing suitable intermediate adsorption energies for CO_2_ reduction, thus integrating the three functions of N_2_ activation, CO_2_ reduction, and C−N coupling [12, 160−164]. Among Pd‐based catalysts, the Pd_1_Cu_1_/TiO_2_ catalyst achieves synergistic performance optimization through oxygen vacancy regulation and alloying [12]. PdCu alloy nanoparticles with a particle size of 5–8 nm were loaded onto an oxygen‐vacancy‐rich TiO_2_ support (Figure 11a). The Ti^3+^ in the support shifted the d‐band center of Pd from −1.2 eV to −0.8 eV via electron transfer, significantly enhancing the side‐on adsorption of N_2_ molecules and elongating the N≡N bond from 1.09 Å to 1.15 Å, substantially lowering the activation energy barrier (Figure 11b). Meanwhile, the Cu sites efficiently catalyze the reduction of CO_2_ to generate *CO by modulating the adsorption strength of the *COOH intermediate. The spatial matching of the two types of active sites enables smooth C–N coupling, achieving a FE of 8.92% and a UYR of 3.36 mmol h^−1^ g_cat_^−1^ (Figure 11c). Ru‐based catalysts have attracted considerable attention due to their outstanding N_2_ activation capability. Meng et al. [160] prepared a Ru_1_–ZrO_2_/C single‐atom catalyst via a molten salt method, which exhibited remarkable performance. This catalyst employed UiO‐66‐derived ZrO_2_ as the support, with Ru single atoms anchored onto the support surface via a molten salt method to form a Ru–O_4_ coordination configuration (Figure 11d). Its unique bifunctional site design, combined with the hydrogen spillover effect, synergistically optimizes the reaction process (Figure 11e–g). The d‐band center of the Ru single‐atom sites, modulated by electronic regulation, enables efficient N_2_ activation, while the cooperative interaction with adjacent Zr sites constructs CO_2_ adsorption and activation centers. Meanwhile, the Ru sites promote water dissociation to generate *H, and the hydrogen spillover effect facilitates rapid migration of *H to the Zr sites, providing sufficient protons for intermediate hydrogenation and effectively lowering the C–N coupling energy barrier. At −0.5 V versus RHE, this catalyst achieves a UYR of 1.56 mmol h^−1^ g^−1^ and a FE of 5.55%, while maintaining a stable current density during 12 h of long‐term electrolysis, demonstrating excellent structural stability (Figure 11h). In addition to Pd‐ and Ru‐based catalysts, Pt‐based modified electrodes have also achieved performance optimization through surface regulation [165]. Pt electrodes modified with polyaniline (PAni) or polypyrrole (PPy) coatings enable urea synthesis at an extremely low overpotential of −0.165 V versus NHE in a high‐pressure system of 60 bar (30 bar N_2_ + 30 bar CO_2_), through modulation of the Pt surface electronic states and proton transport. Among them, the PPy/Pt electrode exhibits superior performance, achieving a urea concentration of 21.2 µM and a FE of 6.5%, with a Tafel slope of 121 mV dec^−1^ consistent with the Volmer‐type hydrogen participation mechanism. The PAni/Pt electrode achieves corresponding values of 8.0 µM and 3.1%. Both coatings enlarge the reaction surface area through their porous structures, promoting the co‐adsorption and activation of N_2_ and CO_2_. However, noble metal‐based catalysts suffer from inherent drawbacks [166, 167, 168, 169, 170, 171]. First, noble metals such as Ru, Pt, and Pd are scarce in reserves and costly, and their synthesis processes are prone to single‐atom aggregation or nanoparticle sintering due to high‐temperature treatment, reducing the density of active sites. Second, the strong adsorption of intermediates such as *CO on noble metal surfaces can lead to active site poisoning, while their hydrogen adsorption characteristics tend to trigger severe HER, which strongly competes with the target C–N coupling reaction, substantially reducing urea selectivity. Third, Pt‐based coated electrodes are susceptible to coating detachment under strong hydrogen evolution at potentials below −0.4 V, and Ru‐based catalysts also suffer from active site leaching during long‐term electrolysis, making their stability insufficient to meet practical application requirements [169, 171]. These issues collectively limit the industrial application of noble metal‐based catalysts and have promoted the development of non‐noble metal‐based catalysts.

**FIGURE 11:**
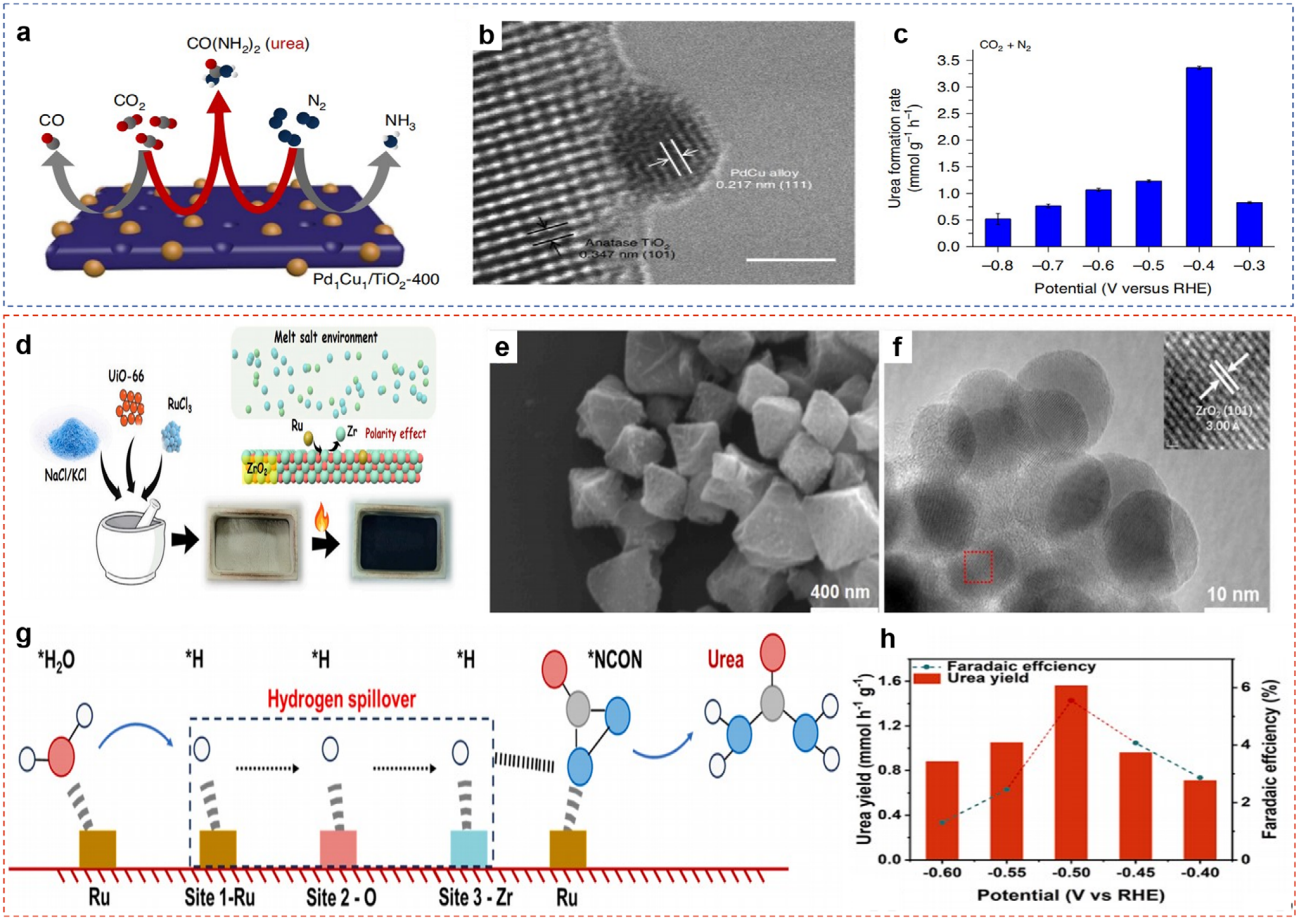
a) Schematic diagram for urea synthesis. The grey, navy, red, and white spheres represent the C, N, O, and H atoms, respectively. b) High‐resolution TEM image of Pd_1_Cu_1_/TiO_2_‐400. The scale bar is 2 nm. c) Urea generation with CO_2_ and N_2_ as feeding gases. Reproduced with permission [12]. Copyright 2020, Springer Nature. d) Schematic of single‐atom loading by the high‐temperature molten salt method. e) SEM image of Ru_1_‐ZrO_2_/C, f) TEM image of Ru_1_‐ZrO_2_/C. g) Schematic of hydrogen spillover phenomenon on Ru_1_‐ZrO_2_/C catalysts. h) Electrocatalytic activity tests of Ru_1_‐ZrO_2_/C catalysts. Reproduced with permission [160]. Copyright 2025, Elsevier.

#### 3.1.2 Non‐Noble Metal‐Based Catalysts

Non‐noble metals (Mo, Co, Fe, Bi, etc.), by virtue of their abundant reserves, low cost, and tunable electronic structures, have become a core research direction for the N_2_ + CO_2_ electrocatalytic urea synthesis system. Their catalytic activity mainly originates from the synergistic interaction between metal atoms and coordinating atoms (O, N, P, etc.), enabling efficient synergy among N_2_ activation, CO_2_ reduction, and C–N coupling through modulation of the d‐band center and optimization of intermediate adsorption energy barriers [147, 172, 173, 174, 175, 176]. Among Mo‐based catalysts, MoP stands out due to its noble‐metal‐like electronic properties [177]. The d‐band center of the Mo sites on the MoP (101) facet was modulated to −1.0 eV, enabling effective electronic overlap with the π* antibonding orbitals of N_2_ molecules, which were subsequently converted to the key N‐containing intermediate *NNH via protonation steps. Meanwhile, CO_2_ is reduced on the catalyst surface to generate the *CO intermediate, which undergoes targeted C–N coupling with *NNH to form *NCON, followed by multi‐step protonation and electron transfer to ultimately yield urea. This catalyst achieves a FE of 36.5%, representing an excellent level among Mo‐based non‐noble metal catalysts reported to date. Co‐based catalysts have attracted considerable attention due to their flexible coordination environments and strong N_2_ activation capability [178]. In the Co–PMDA–2mbIM conductive metal–organic framework (MOF) catalyst, Co atoms coordinated with six O atoms to form CoO_6_ octahedral active centers (Figure 12a). The 2‐methylbenzimidazole guest molecules served as charge transfer bridges. These bridges significantly enhanced charge transport efficiency within the framework, while simultaneously strengthening the adsorption capacities for N_2_ and CO_2_, effectively suppressing intermediate desorption (Figure 12b). This catalyst achieves a UYR of 14.47 mmol h^−1^ g_cat_^−1^ and a FE of 48.97%, demonstrating excellent activity and selectivity (Figure 12c). Fe‐based catalysts, relying on structural designs such as phthalocyanine complexes and dual sites, have also demonstrated promising performance. The iron phthalocyanine (FePc) catalyst, by virtue of its unique molecular structure, not only suppresses the competitive HER but also synergistically activates N_2_ and CO_2_. In 0.1 M KHCO_3_ electrolyte, this catalyst exhibits optimal performance at −0.4 V versus RHE, achieving a UYR of 357 µmol h^−1^ g_cat_^−1^ and a FE of 14.36%, while maintaining stable activity after 5 cycles [179]. In addition, Bi‐based heterostructure catalysts optimize charge transfer efficiency through interface engineering. In the Bi–BiVO_4_ Mott–Schottky heterostructure, a space charge region forms at the Bi/BiVO_4_ interface, enabling spontaneous directional charge transfer from BiVO_4_ to Bi (Figure 12d–f), which modulates the electronic states of Bi sites to enhance N_2_ and CO_2_ adsorption and activation, achieving a FE of 12.55% and a UYR of 5.91 mmol h^−1^ g^−1^ (Figure 12g). The BiFeO_3_/BiVO_4_ p–n heterojunction, leveraging its built‐in electric field to promote interfacial charge separation, synergistically activates N_2_ and CO_2_, further increasing the FE for urea to 17.18%. This provides diverse design concepts for heterostructured non‐noble metal catalysts (Figure 12h) [180].

**FIGURE 12:**
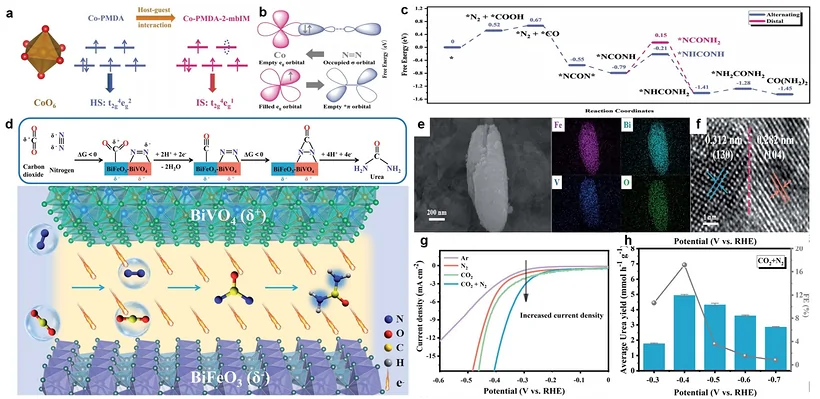
a) A schematic illustration of spin‐state regulation in Co–PMDA–2‐mbIM induced by host–guest interaction. b) A simplified schematic diagram of N_2_ bonding to a Co center. c) The possible urea electrosynthesis mechanism. Reproduced with permission [178]. Copyright 2022, Royal Society of Chemistry. d) The schematic electrocatalytic urea production mechanism based on BiFeO_3_/BiVO_4_ p–n heterostructure synergistic effects. e) SEM image and the corresponding elemental mapping of BiFeO_3_/BiVO_4_ hybrids. f) High‐resolution TEM image of BiFeO_3_/BiVO_4_ hybrids, and the dotted line represents the heterointerfaces. g) LSV curves of BiFeO_3_/BiVO_4_ hybrids in Ar, N_2_, CO_2_ and N_2_ + CO_2_ saturated electrolyte. h) The urea yield rate and faradaic efficiencies for urea production with N_2_ and CO_2_ as the feed gas at various potentials for BiFeO_3_/BiVO_4_ hybrids. Reproduced with permission [180]. Copyright 2021, Royal Society of Chemistry.

#### 3.1.3 Frustrated Lewis Pairs (FLPs)

As a unique class of catalytic systems in which steric hindrance prevents direct bond formation between Lewis acid and base sites, FLPs provide innovative concepts for the co‐activation of N_2_ and CO_2_ and the C–N coupling synthesis of urea. By virtue of their integrated “adsorption–activation–coupling” active site design, they effectively overcome the bottlenecks of conventional catalysts, including weak adsorption of inert gas molecules, high activation energy barriers, and low coupling selectivity. The core catalytic mechanism lies in the targeted capture of N_2_ (electron‐rich) and CO_2_ (electron‐deficient) through the empty orbitals of Lewis acid (LA) sites and the lone‐pair electrons of Lewis base (LB) sites, followed by molecular activation and directed coupling via orbital interactions. The interaction of LA sites with the bonding orbitals of gas molecules and LB sites with the antibonding orbitals weakens the N≡N and C = O bond strengths, laying the foundation for subsequent C–N coupling [181, 182, 183, 184, 185]. In a specific catalytic system, Ni_3_(BO_3_)_2_ nanocrystals construct FLP active sites through low‐temperature annealing, in which unsaturated Ni sites serve as LA and adjacent hydroxyl groups serve as LB (Figure 13a,b) [148]. The synergistic interplay between the two endows the catalyst with excellent adsorption capabilities for both N_2_ and CO_2_. The annealing treatment also induces a transition of Ni ions from a high‐spin to a low‐spin state, and the empty e_g_ orbitals promoted the directed coupling of N = N and CO intermediates through a “σ‐orbital carbonylation” strategy, generating the key precursor *NCON (Figure 13c–e). At −0.5 V versus RHE, this catalyst achieves a UYR of 9.70 mmol h^−1^ g_cat_^−1^ and a FE of 20.36%, with no significant performance decay after 20 h of long‐term electrolysis (Figure 13f,g). Similarly, InOOH nanocrystals construct In–OH–In‐type FLPs through regulation of annealing temperature [186]. The electron‐deficient In sites (LA) and electron‐rich In–OH sites (LB) target the adsorption of N_2_ and CO_2_, respectively, achieving efficient activation of reactant molecules via orbital interactions (Figure 13h–j). The optimal sample, InOOH‐100, achieved a UYR of 6.85 mmol h^−1^ g_cat_^−1^ and a FE of 20.97% at −0.4 V versus RHE (Figure 13k,m). SCN^−^ poisoning experiments confirmed that the synergy between LA and LB sites is the core of catalytic activity. This class of FLP‐based catalysts, without the need for noble metal components, achieves efficient urea synthesis under ambient conditions through the synergistic design of non‐noble metal sites and surface functional groups. They not only resolve the challenge of balancing activity and selectivity faced by conventional catalysts but also offer advantages of low cost and high stability, providing a novel paradigm for active site design in electrocatalytic urea synthesis and offering important inspiration for catalyst development in other inert small‐molecule conversion reactions [20, 187, 188, 189, 190].

**FIGURE 13:**
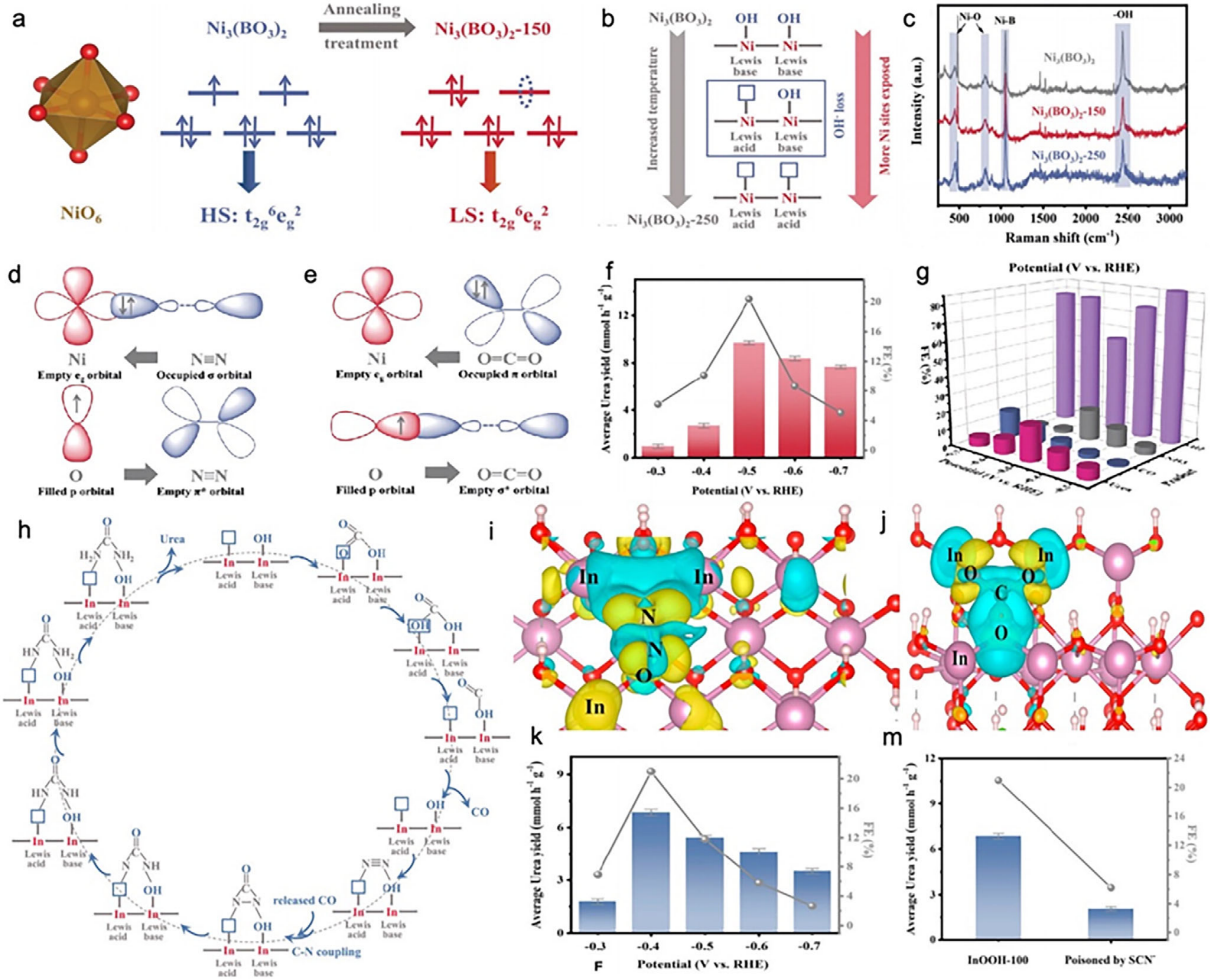
a) Schematic illustration of the spin‐state regulation of pristine Ni_3_(BO_3_)_2_ after annealing treatment. b) Schematic illustration of the formation of frustrated Lewis pair sites affected by the calcination temperature. c) Raman spectra of the pristine Ni_3_(BO_3_)_2_, Ni_3_(BO_3_)_2_‐150, and Ni_3_(BO_3_)_2_‐250 catalysts. Schematic illustration of the charge “donation–acceptance” process between d) frustrated Lewis pairs and N_2_ molecule; e) frustrated Lewis pairs and CO_2_ molecule. f) Urea yield rate and Faradaic efficiencies and g) corresponding product distribution of H_2_, CO, NH_3_, and urea with N_2_ and CO_2_ as the feed gas at various potentials for the Ni_3_(BO_3_)_2_‐150 catalysts. Reproduced with permission [148]. Copyright 2021, Royal Society of Chemistry. h) Schematic illustration of the electrocatalytic co‐activation of N_2_ and CO_2_ into urea over artificial FLPs of InOOH‐100 catalysts. The charge density difference of i) N_2_ and j) CO_2_ adsorbed on FLPs sites; the yellow and cyan color indicate electron accumulation and depletion, respectively, with an isosurface value of 0.013 e Å^−3^. k) The urea yield rate and Faradaic efficiencies of InOOH‐100 catalysts. m) Average urea yield of InOOH‐100 and InOOH‐100 poisoned by SCN^−^. Reproduced with permission [186]. Copyright 2021, Elsevier.

#### 3.1.4 Mechanistic Challenges and Breakthroughs

##### 3.1.4.1 C–N Coupling Mechanism

The C–N coupling mechanism for the electrocatalytic synthesis of urea from N_2_ and CO_2_ is centered around the “directed reaction between N‐containing and C‐containing intermediates.” Based on the differences in the timing of *CO insertion and the types of N‐containing intermediates, it is mainly divided into two core pathways, both of which are characterized by the formation of the key intermediate *NCON as the core feature (Figure 14a,b) [12, 191, 192, 193, 194, 195, 196].

**FIGURE 14:**
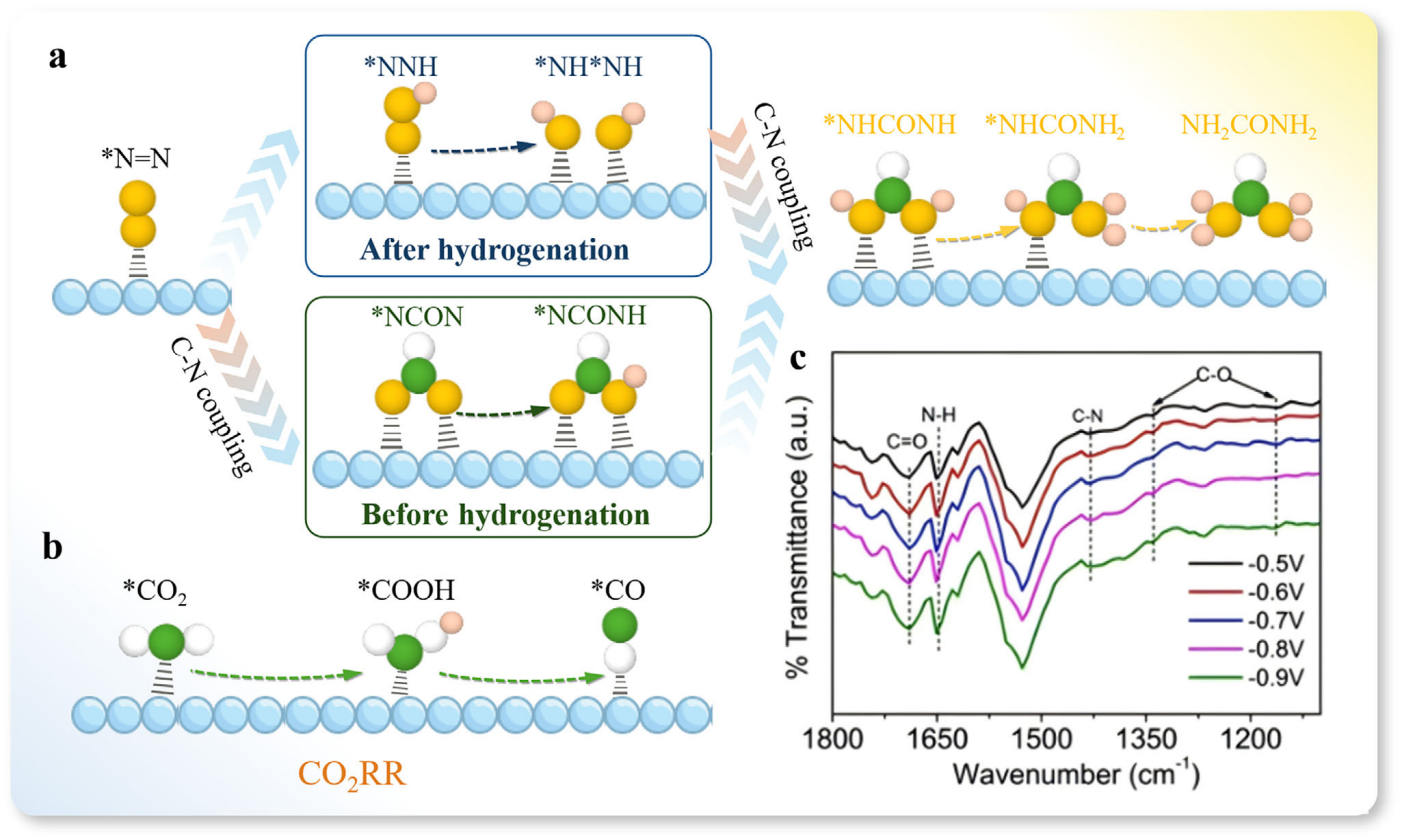
a) and b) The reaction pathway of CO_2_ and N_2_ coupling to synthesize urea. c) ATR‐FTIR spectroscopy experiments of the electrolytes at different potentials during electrocatalytic co‐reduction of N_2_ and CO_2_ using CoPc–MoS_2_. Reproduced with permission [194]. Copyright 2023, Royal Society of Chemistry.

##### 3.1.4.2 Pre‐Hydrogenation Coupling Pathway (NCON Pathway)

This pathway is the most thermodynamically and experimentally validated mainstream route. CO_2_ first underwent reduction on the catalyst surface via the *CO_2_ → *COOH → *CO process to generate the C‐containing intermediate CO, in which the formation of *COOH was the rate‐determining step (RDS) of the overall reaction [12, 195]. Meanwhile, N_2_ molecules are adsorbed and activated by the active sites of the catalyst, with the N≡N bond weakened to form the *N = N intermediate. The unhydrogenated N = N then undergoes directed insertion coupling with CO to generate the tower‐shaped key intermediate *NCON. In situ Fourier‐transform infrared spectroscopy has detected characteristic vibrational peaks of *NCON at 1690 cm^−1^ (C = O) and 1431 cm^−1^ (C–N) (Figure 14c) [194]. Subsequently, *NCON undergoes four consecutive protonation and hydrogenation steps, sequentially converting to *NCONH, *HNCONH, and *HNCONH_2_, ultimately yielding urea [193].

##### 3.1.4.3 Post‐Hydrogenation Coupling Pathway

In this pathway, the activation and hydrogenation of N_2_ occur prior to C–N coupling. After N_2_ is adsorbed and activated to form N = N, it first undergoes two‐step PCET hydrogenation, cleaving the N–N bond to generate the *NHNH intermediate. Subsequently, the *CO generated from CO_2_ reduction inserts into *NHNH, undergoing an exothermic C–N coupling reaction to form the *NHCONH intermediate, which is then converted to urea through two further hydrogenation steps [192]. In addition, theoretical calculations indicated that CO can also couple with N‐containing hydrogenated intermediates such as *NNH and *NH_2_ generated during the hydrogenation of N = N, forming intermediates such as *NCONH and *NH_2_CONH, all of which ultimately completed urea synthesis through protonation [193].

Between the two pathways, the NCON pathway is the dominant route followed by most efficient catalysts, due to its structurally stable intermediates, fewer competing side reactions, and lower energy barriers for subsequent protonation steps (distal pathway ΔG = 0.54 eV) [180, 191, 197].

#### 3.1.5 Bottlenecks of C–N Coupling

C–N coupling is the efficiency‐controlling step in the N_2_ + CO_2_ electrocatalytic synthesis of urea, with its core bottlenecks concentrating on four major aspects: insufficient synergy of intermediates, limited catalyst functionality, interference from competitive reactions, and mass transfer supply imbalance, which directly constrain the yield and selectivity of urea. The generation rates of N‐containing intermediates (*N = N, *NHNH) and C‐containing intermediates (CO) exhibited significant asynchrony (the energy barrier for *CO formation is 0.5–1.0 eV, lower than the 0.8–1.2 eV for N = N activation), and N = N was prone to desorption loss while *NHNH may undergo excessive hydrogenation to NH_3_, resulting in reduced concentrations of effective intermediates [12, 146, 198]. Conventional catalysts with single‐function active sites struggle to simultaneously meet the multi‐step requirements of N_2_ activation, CO_2_ reduction, and C–N coupling, lacking multifunctional sites capable of synergistically regulating the adsorption of both gases and the migration of intermediates. During the PCET process, the HER (energy barrier of 0.3–0.8 eV), along with parallel side reactions such as excessive reduction of N_2_ to NH_3_ and reduction of CO_2_ to CO/HCOOH, fiercely competes for electrons, protons, and reactants, weakening coupling selectivity [173, 199]. Moreover, the extremely low solubility of N_2_ in aqueous electrolytes at ambient temperature (approximately 0.03 mol L^−1^), slow gas diffusion, and high interfacial mass transfer resistance lead to insufficient N_2_ supply on the catalyst surface, creating an imbalance of “carbon source excess and nitrogen source deficiency,” which further constrains C–N coupling efficiency [200, 201].

To address the aforementioned N_2_ activation challenges and C–N coupling bottlenecks, current research has achieved breakthroughs through three core strategies: multifunctionalization of active sites, regulation of intermediate adsorption, and optimization of the reaction environment. Multifunctional structures such as FLPs and dual‐atom sites are designed to synergistically enhance the co‐adsorption and activation of N_2_ and CO_2_. The electronic states of metal centers (e.g., d‐band center, e_g_ orbital occupancy) are modulated to optimize intermediate adsorption strengths and match the generation rates of intermediates. Combined with electrolyte modification and reactor innovation, HER and parallel side reactions are suppressed, significantly enhancing C–N coupling selectivity and efficiency, thereby providing key technological pathways for efficient electrocatalytic urea synthesis.

### 3.2 Current Performance Status and Gap

Compared with the NO_3_^−^ + CO_2_ system, the performance of the N_2_ + CO_2_ system still exhibits a significant gap. Table 2 summarizes several representative catalysts for this system in recent years.

**TABLE 2:** Representative performance metrics of N_2_ + CO_2_ electrocatalytic urea synthesis.

Catalyst	Electrolyte	Potential (vs. RHE)	Current density	FE	Original UYR (Reactor)	Unified UYR	Stability	Refs
Pd_1_Cu_1_/TiO_2_‐400 (Precious metals)	0.1 M KHCO_3_ (N_2_+CO_2_ saturation)	−0.4 V	≈ −4 mA cm^−2^	8.92%	3.36 mmol·h^−1^·g_cat_^−1^ (GDE)	3.36 mmol·h^−1^·g_cat_^−1^	6 cycles	[12]
Ru_1_‐ZrO_2_/C (Precious metals)	0.1 M KHCO_3_ (N_2_+CO_2_ saturation)	−0.5 V	≈ −0.2 mA cm^−2^	5.55%	1.56 mmol·g^−1^·h^−1^ (H‐cell)	1.56 mmol·h^−1^·g_cat_^−1^	12 h	[160]
PPy/Pt (Precious metals)	0.1 M Li_2_SO_4_/0.03 M H^+^ (N^2^+CO_2_ saturation)	−0.325 V (NHE)	≈ −0.07 mA cm^−2^	5.7%	21.2 µmol/5 h (H‐cell)	4.24 µmol h^−1^(not convertible to per g)	5 h	[165]
PAni/Pt (Precious metals)	0.1 M Li_2_SO_4_/0.03 M H^+^ (N_2_+CO_2_ saturation)	−0.325 V (NHE)	≈ −0.05 mA cm^−2^	4.8%	12.0 µmol/5 h (H‐cell)	2.40 µmol h^−1^(not convertible to per g)	5 h	[165]
MoP (Non‐precious metals)	0.1 M KHCO_3_ (N_2_+CO_2_ saturation)	−0.35 V	≈ −2 mA cm^−2^	36.5%	12.4 µg·h^−1^·mg_cat_^−1^ (H‐cell)	0.206 mmol·h^−1^·g_cat_^−1^	5 cycles	[177]
Co‐PMDA‐2mbIM (MOF)	0.1 M KHCO_3_ (N_2_+CO_2_ saturation)	−0.5 V	≈ −4 mA cm^−2^	48.97%	14.47 mmol·h^−1^·g^−1^ (H‐cell)	14.47 mmol·h^−1^·g_cat_^−1^	20 h	[178]
FePc (Non‐precious metals)	0.1 M KHCO_3_ (N_2_+CO_2_ saturation)	−0.4 V	≈ −2 mA cm^−2^	14.36%	357 µmol·h^−1^·g_cat_^−1^ (H‐cell)	0.357 mmol·h^−1^·g_cat_^−1^	5 cycles	[179]
BiFeO_3_/BiVO_4_ (Mott‐Schottky)	0.1 M KHCO_3_ (N_2_+CO_2_ saturation)	−0.4 V	≈ −3 mA cm^−2^	12.55%	5.42 mmol·h^−1^·g^−1^ (H‐cell)	5.42 mmol·h^−1^·g_cat_^−1^	10 h	[180]
Ni_3_(BO_3_)_2_‐150 (FLPs)	0.1 M KHCO_3_ (N_2_+CO_2_ saturation)	−0.5 V	≈ −4 mA cm^−2^	20.36%	9.70 mmol·h^−1^·g_cat_^−1^ (H‐cell)	9.70 mmol·h^−1^·g_cat_^−1^	20 h	[148]
InOOH‐100 (FLPs)	0.1 M KHCO_3_ (N_2_+CO_2_ saturation)	−0.4 V	≈ −3 mA cm^−2^	20.97%	6.85 mmol·g^−1^·h^−1^ (H‐cell)	6.85 mmol·h^−1^·g_cat_^−1^	20 h	[186]

Similarly, the urea FE for the N_2_ + CO_2_ system should be clarified separately, because the electron‐transfer stoichiometry differs from that of the NO_3_^−^ + CO_2_ system. Under the ideal full‐reduction pathway, the overall reaction involves 6 electrons per urea molecule:

(3) $$N_{2}+CO_{2}+6H^{+}+6e^{-}\to \mathrm{CO}{\left(NH_{2}\right)}_{2}+H_{2}O$$

The FE is therefore calculated as:

(4) $$F_{\mathrm{urea}}\left(\%\right)=\frac{6\times F\times C_{\mathrm{urea}}\times V}{M_{\mathrm{urea}\;}\times Q}\times 100\%$$

where the symbols have the same meaning as in the NO_3_^−^ + CO_2_ system [12, 147]. In practice, however, the electron consumption is often higher than the ideal 6 e^−^ due to competing hydrogen evolution and side reactions, and some studies may adopt different electron‐transfer assumptions depending on the proposed mechanism [135, 199]. Therefore, the FE values reported for the N_2_ + CO_2_ system should be compared only when the same electron‐transfer assumption and quantification method are used.

Among the representative catalysts for the N_2_ + CO_2_ electrocatalytic urea synthesis system, the conductive MOF (Co–PMDA–2mbIM) exhibits the best performance, with a FE of 48.97% and a yield rate significantly superior to those of FLP‐based, heterojunction, conventional metal‐based, and metal phthalocyanine‐based catalysts, primarily attributed to the precise regulation and synergistic effects of the electronic states of active sites. All types of catalysts demonstrate good long‐term electrolysis stability by virtue of their stable structural frameworks or crystalline phases. However, the overall performance of this system still exhibits a significant gap compared with the NO_3_^−^ + CO_2_ system, in which the optimal FE and yield rate are far higher. The root cause lies in the N≡N bond energy of N_2_ (940.95 kJ mol^−1^) being much higher than the N = O bond dissociation energy of NO_3_^−^ (204 kJ mol^−1^), combined with the extremely low solubility of N_2_ in electrolyte (≈0.03 mol L^−1^), leading to high activation barriers and insufficient reactant supply. Meanwhile, the adsorption strengths and generation rates of N_2_ reduction intermediates (*N, *NNH) and CO_2_ reduction intermediates (*CO, *COOH) are difficult to match, reducing the probability of C–N coupling, with more severe competition from HER. In contrast, NO_3_^−^ can be rapidly reduced to *NO and *NH_2_ with favorable potential matching with CO_2_RR, and catalyst design is more adaptable, further amplifying the performance gap between the two systems.

Despite the rapid progress achieved in catalyst development and mechanistic understanding, the reliability of reported urea production rates and Faradaic efficiencies in the N_2_ + CO_2_ system requires careful evaluation. Unlike nitrate‐based systems, where the nitrogen source is introduced as a well‐defined ionic reactant, N_2_ is chemically inert, and its electrochemical activation involves highly diluted nitrogen‐containing intermediates. Consequently, trace nitrogen‐containing contaminants originating from electrolytes, gas sources, catalyst precursors, ion‐exchange membranes, or the surrounding environment may interfere with urea detection, particularly when the product concentration is extremely low. Similar challenges have been widely recognized in electrochemical nitrogen reduction reactions, where rigorous isotope‐labeling protocols and contamination control procedures were demonstrated to be essential for reliable nitrogen‐source verification [114, 202, 203, 204].

To establish the authentic origin of nitrogen and carbon atoms in the synthesized urea, dual‐isotope labeling experiments using ^15^N_2_ and ^13^CO_2_ combined with nuclear magnetic resonance (NMR) spectroscopy or mass spectrometry (MS) analysis should be considered a critical validation requirement. The simultaneous identification of ^15^N‐ and ^13^C‐containing urea can directly confirm that the product originates from the supplied N_2_ and CO_2_ feedstocks rather than from adventitious nitrogen‐containing impurities. In addition, comprehensive control experiments, including open‐circuit potential measurements, Ar atmosphere controls, electrolyte purification, and quantitative determination of background NH_3_/NO_x_^−^ species, are highly recommended to exclude possible contamination pathways [12, 147, 148, 149, 202, 204].

However, a critical assessment of the high‐performance N_2_ + CO_2_ electrosynthesis systems summarized in Table 2 indicates that such comprehensive validation procedures have not yet been universally implemented. Some studies mainly rely on urea detection, yield rate, and Faradaic efficiency measurements, while lacking systematic isotope‐labeling verification or detailed impurity analysis. Therefore, although these studies provide valuable insights into catalyst design, intermediate regulation, and C–N coupling chemistry, the proposed reaction pathways and quantitative performance evaluations should be interpreted with appropriate caution. Recent advances in operando characterization techniques, including differential electrochemical mass spectrometry (DEMS), in situ Raman spectroscopy, and isotope‐assisted analysis, have provided more direct evidence for nitrogen intermediate evolution and C–N coupling processes, representing an important direction for improving mechanistic reliability [109, 110, 111, 194, 205]. Moving forward, standardized evaluation protocols integrating isotope tracing, blank controls, impurity quantification, and operando characterization will be indispensable for establishing reliable benchmarks and accelerating the practical development of N_2_ + CO_2_ electrocatalytic urea synthesis.

## 4 System Comparison and Application Suitability

The preceding sections have respectively reviewed the research progress of the NO_3_^−^ + CO_2_ and N_2_ + CO_2_ systems in terms of catalyst design, reaction mechanisms, and performance optimization. Although both systems ultimately achieve C–N bond construction for urea molecules via electrochemical means, their essence is not simply “two feedstock alternatives for the same reaction,” but rather two technological routes built upon fundamentally different nitrogen source activation difficulties, interfacial mass transfer behaviors, and reaction network complexities. The former is closer to “pollutant resource utilization coupled with carbon conversion,” while the latter represents the ultimate green route of “direct urea synthesis from air and CO_2_.” Therefore, comparing solely on the basis of a single metric such as Faradaic efficiency or yield rate fails to capture the true differences between the two systems. A more meaningful approach is to conduct a systematic comparison from dimensions including feedstock intrinsic properties, reaction kinetic constraints, catalyst design objectives, reactor coupling requirements, and industrial applicability scenarios.

Overall, the reason why the NO_3_^−^ + CO_2_ system exhibits higher Faradaic efficiency, higher urea yield rate, and faster engineering advancement at the current stage lies fundamentally in the fact that NO_3_^−^ itself is already in a relatively high oxidation state, possessing both higher aqueous availability and a lower reduction activation barrier, which enables nitrogen‐containing intermediates to form more readily at the catalytic interface [17, 18, 206]. In contrast, the N_2_ + CO_2_ system must first overcome the strong inertness of the N≡N triple bond, followed by addressing a series of issues including low solubility, low surface coverage, and easy loss of intermediates. Therefore, its primary challenge is no longer simply “how to improve coupling selectivity,” but rather “how to truly enable N_2_ to enter the coupling reaction network under ambient temperature and pressure.” [207] This difference dictates that the two systems should not be viewed as simple competitors at the current stage, but rather as functionally complementary routes, with one being a near‐term deployable route and the other a long‐term strategic goal [208].

### 4.1 Feedstock Properties and Thermodynamic/Kinetic Differences

The most fundamental difference between the two systems originates from the chemical nature of the nitrogen source molecules [8]. The N_2_ molecule possesses extremely high bond energy and very low polarizability, with an N≡N bond energy as high as 941 kJ mol^−1^ and extremely low solubility in aqueous solution, rendering its adsorption, activation, and subsequent protonation on the electrode surface highly challenging [8, 209, 210]. In contrast, the N–O bond dissociation in NO_3_^−^ is significantly less difficult, and nitrate itself exhibits high solubility and favorable mass transfer accessibility in the aqueous phase, enabling sustained supply of nitrogen‐containing precursors to the interface over a wide potential window [179, 209, 211]. Therefore, from the feedstock perspective, the NO_3_^−^ + CO_2_ system essentially belongs to “synergistic reduction involving a readily activatable nitrogen source,” while the N_2_ + CO_2_ system belongs to “coupling conversion driven by deep activation of inert molecules.” This distinction is not merely a matter of degree, but a structural difference that determines the upper limit of the entire reaction pathway.

From the perspective of thermodynamic and kinetic constraints, the rate‐controlling steps of the two systems are also distinctly different. For the NO_3_^−^ + CO_2_ system, the research focus primarily rests on the rate matching between the two half‐reactions of NO_3_^−^RR and CO_2_RR, as well as the effective coupling of key N‐containing and C‐containing intermediates at adjacent sites [101, 212]. That is to say, its main challenge is a “synergy problem” rather than a “whether it can be initiated” problem. In contrast, in the N_2_ + CO_2_ system, the N_2_ → *NNH or N_2_ → activated adsorption state is often the most difficult step in the entire reaction network, with the rate‐determining step frequently shifting to the very initial stage of the reaction. As a result, even if CO_2_ can readily generate C‐containing intermediates such as *CO in subsequent steps, efficient C–N coupling remains difficult to achieve due to the lack of sufficiently effective N‐containing intermediates [176, 210, 213]. In other words, the bottleneck of the NO_3_^−^ + CO_2_ system leans more toward multi‐intermediate synergistic regulation, whereas the bottleneck of the N_2_ + CO_2_ system is first and foremost the insufficient reactivity of the nitrogen source.

In addition, the two systems also differ in terms of electron economy and side‐reaction competition patterns. The NO_3_^−^ + CO_2_ route generally requires a lower total number of electron transfer steps, and the pathways for generating N‐containing intermediates are relatively well‐defined, making it easier to shorten the deep reduction chain through site engineering [108, 118, 128, 214]. In contrast, the N_2_ + CO_2_ route not only involves more electron transfer steps, but also exhibits substantial overlap between the N_2_ activation process and the HER in terms of potential windows, making it highly prone to competitive diversion of electrons and protons. Particularly in aqueous electrolytes, if the active sites exhibit excessively strong adsorption of *H, the system tends to preferentially proceed toward HER rather than N_2_ activation, which is the fundamental reason why many N_2_ + CO_2_ systems, despite having theoretically feasible pathways, still yield only low urea selectivity experimentally [175, 215].

Based on the above differences, the performance upper limits of the two systems exhibit a clear stratification at the current stage. The NO_3_^−^ + CO_2_ system has already achieved Faradaic efficiencies approaching 90% and urea yield rates on the order of hundreds of mmol h^−1^ g_cat_^−1^ or even higher on selected catalysts and in intensified reactors, whereas the best results of the N_2_ + CO_2_ system remain largely confined to the low‐to‐medium yield and moderate selectivity ranges. This gap is not merely a matter of different degrees of catalyst development, but rather a profound reflection of the intrinsic differences between the two feedstock systems in terms of molecular activation difficulty, interfacial coverage, and kinetic accessibility [64, 174, 216, 217]. To more systematically compare the differences between the NO_3_^−^ + CO_2_ and N_2_ + CO_2_ systems in terms of feedstock characteristics, reaction mechanisms, and engineering suitability. Table 3 summarizes the key comparative dimensions of the two systems.

**TABLE 3:** Comparative analysis of NO_3_^−^ + CO_2_ and N_2_ + CO_2_ electrocatalytic urea synthesis.

Category	NO_3_^−^ + CO_2_ system	N_2_ + CO_2_ system
Nitrogen source nature	High solubility, pre‐activated	Chemically inert, low solubility
Key kinetic limitation	Reaction rate matching (NO_3_^−^RR vs CO_2_RR)	N_2_ activation barrier
Typical intermediates	*NO_2_, *NO, *NH_2_	*NNH, *NHNH
C–N coupling pathways	Multiple (e.g., *CO–*NH_2_, *CO–*NO)	Limited (*NCON‐based)
Catalyst design principle	Dual‐site synergy & side‐reaction suppression	N_2_ activation + intermediate stabilization
Role of multi‐sites	Kinetic matching	Kinetic matching
Reactor requirement	Enhance CO_2_ mass transfer	Dual‐gas transport & enrichment
Typical FE (state‐of‐art)	Up to ∼90%	< 50%
Typical yield	up to hundreds mmol·h^−1^·g^−1^	< 20 mmol·h^−1^·g^−1^
Technological maturity	Near‐term scalable	Early‐stage
Application scenario	Wastewater denitrification + CO_2_ utilization	Green distributed synthesis
Development priority	Short‐term deployment	Long‐term strategic goal

As shown in Table 3, the two systems exhibit essential differences in terms of kinetic limitations, key intermediate types, catalyst design logic, and reactor requirements, and these differences will be further discussed in the subsequent sections.

### 4.2 Differences in Key Intermediate Formation and C–N Coupling Modes

If further compared from the perspective of the reaction network, the differences between the two systems can be summarized as follows: the NO_3_^−^ + CO_2_ system resembles “multiple N‐containing intermediate branches converging toward the same coupling center,” while the N_2_ + CO_2_ system resembles “restricted coupling driven by very few effective N‐containing intermediates.” In the former, NO_3_^−^ can be gradually reduced on the electrode surface to generate a variety of intermediates including *NO_2_, *NO, *NH_2_OH, *NH_2_, and *NH, meaning that catalysts can select nodes more suitable for direct coupling with *CO or CO_2_ by modulating the depth of reduction [28, 205]. Consequently, multiple possible pathways have emerged in the NO_3_^−^ + CO_2_ system, including *CO–*NH_2_, *CO–*NO, *CO–*NO_2_, and even direct coupling between CO_2_ and N‐containing intermediates [216, 218, 219]. Although the mechanistic details remain under debate, this at least indicates that this system possesses a relatively high “redundancy” in terms of intermediate types, thereby leaving more room for catalyst design regulation.

The N_2_ + CO_2_ system, however, is different. Its effective N‐containing intermediates are mainly limited to a few species such as the activated *N_2_ state, *NNH, *NHNH, and *NH_2_, and the formation of these intermediates is inherently difficult with short lifetimes [16, 180]. The direct consequence is that C–N coupling in the N_2_ + CO_2_ system does not merely require “finding a low‐energy‐barrier pathway,” but also necessitates the sustained formation of sufficiently concentrated coupling‐capable N‐containing species at the interface. For this reason, most effective pathways in this system currently revolve around *NCON or its derivatives, with the reaction network being relatively convergent and pathway selection more constrained by the electronic states and local configurations of active sites [52, 161, 176]. This “pathway convergence,” on one hand, facilitates theoretical calculations and mechanistic simplification; on the other hand, it also indicates that this system is more strongly dependent on the catalyst microstructure, and any slight deviation may lead to byproduct channels such as NH_3_, CO, or H_2_.

From the perspective of intermediate coupling probability, the advantage of the NO_3_^−^ + CO_2_ system is also reflected in spatiotemporal matching. NO_3_^−^ is present in the electrolyte at high concentrations with rapid diffusion, and although CO_2_ has limited solubility in the aqueous phase, its supply can still be enhanced through means such as GDEs and flow cells [29, 212]. Therefore, as long as the catalytic sites can simultaneously stabilize both C‐containing and N‐containing intermediates, there is a relatively high probability of achieving surface coupling. In the N_2_ + CO_2_ system, however, both feedstocks are gas‐phase molecules, with N_2_ being particularly inert. It is often observed that *CO forms first while the supply of N‐containing intermediates lags behind, resulting in C–N coupling being limited by “scarcity of N intermediates” rather than “insufficiency of C intermediates.” [176, 220, 221] This is also why the N_2_ + CO_2_ system frequently requires structures such as dual sites, heterointerfaces, FLPs, or conductive MOFs to actively enhance the local activation and retention capability of N_2_, thereby artificially establishing conditions for the synchronous generation of intermediates.

### 4.3 Catalyst Design Logic Divergence: Rate Matching vs. N_2_ Activation Priority

Based on the above differences in feedstocks and reaction networks, the catalyst design logic for the two systems is also distinctly divergent [222, 223]. For the NO_3_^−^ + CO_2_ system, the primary goal of catalyst design is typically not simply to enhance the absolute activity of a single step, but rather to establish a dynamic balance between NO_3_^−^RR and CO_2_RR. If NO_3_^−^ reduction proceeds too rapidly while CO_2_ activation is insufficient, N‐containing byproducts such as NH_3_ and NH_2_OH tend to accumulate; conversely, if CO_2_ reduction is too strong while the NO_3_^−^ pathway lags, the reaction may favor C‐containing byproducts such as CO or formic acid. Therefore, the optimal catalysts for this system often feature dual‐site or multi‐site synergistic characteristics, with one type of site favoring NO_3_^−^ activation and N‐containing intermediate generation, and the other type favoring CO_2_ activation and *CO stabilization, while maintaining a moderate spatial proximity between the two to enhance the probability of coupling [224, 225, 226, 227]. In other words, the core design principle of this system can be summarized as: synchronous driving of dual reactions, adjacent‐site coupling of key intermediates, and overall suppression of side reactions.

In contrast, the catalyst design priority for the N_2_ + CO_2_ system must be shifted forward. Since N_2_ itself is difficult to activate, any structural optimization that deviates from “enhancing N_2_ activatability” can hardly bring fundamental performance improvements. Therefore, this system places greater emphasis on reducing the N≡N activation energy barrier through electronic structure engineering, coordination environment modulation, vacancy defect construction, built‐in electric fields, or FLP site design. Only under this premise do catalysts further pursue the stabilization and subsequent coupling of key intermediates such as *NNH, *NHNH, or *NCON. Consequently, a more typical design logic for the N_2_ + CO_2_ system is: N_2_ activation priority → intermediate steady‐state maintenance → synergistic adsorption of C‐ and N‐containing species → coupling pathway guidance [176, 186, 210]. This is also why materials such as noble metal single atoms, noble‐metal‐like MoP, conductive MOFs, and FLPs are particularly valued in this system, as they often simultaneously provide stronger orbital interactions and more precise local electronic modulation capabilities.

Further comparison reveals that although both systems emphasize dual sites and interface engineering, the “reason for needing dual sites” is not the same. In the NO_3_^−^ + CO_2_ system, dual sites are essentially employed to divide the labor in handling two already feasible reduction branches. In the N_2_ + CO_2_ system, however, dual sites serve more to compensate for the functional deficiency that a single site cannot simultaneously accomplish N_2_ activation and intermediate coupling. The former represents “synergistic optimization,” while the latter is closer to “functional compensation.” [228, 229] Therefore, when evaluating catalysts, the same criteria should not be applied to the two systems. For the NO_3_^−^ + CO_2_ system, greater attention should be paid to selectivity, long‐term stability, and performance retention under high current densities. For the N_2_ + CO_2_ system, the primary considerations should be the reliability of isotopic evidence, the genuine extent of N_2_ participation, and whether effective urea generation can be maintained after excluding contamination interference.

### 4.4 Performance Evaluation, Data Comparability, and Limitations

The comparison between the two systems also involves an often‐overlooked issue, namely the comparability boundaries of the data themselves. The normalization methods for urea yield rates in the current literature are not unified, potentially being based on total catalyst mass, metal mass, geometric area, electrode area, or specific reactor volume, making it difficult to directly rank the absolute values across different studies [135]. Similarly, Faradaic efficiency is strongly influenced by reactor configuration, electrolyte composition, nitrogen source concentration, CO_2_ supply mode, and quantitative analysis methods, and cannot be simply compared without considering the testing conditions [207, 222]. Therefore, for a systematic evaluation of the two systems, a more reasonable approach is to compare performance ranges and structural patterns, rather than focusing solely on “whose numerical value is the highest.”

As shown in Figure 15, the NO_3_^−^ + CO_2_ system exhibits a broad current density distribution, spanning from approximately −0.3 mA cm^−2^ (Au@cpCu_7_CF) to approximately −95 mA cm^−2^ (OL‐Cu), with several systems operating in the −10 to −30 mA cm^−2^ range [17, 51, 52, 59, 64, 80, 84]. In sharp contrast, the N_2_ + CO_2_ system is confined to a much narrower and lower range, with most systems operating below −5 mA cm^−2^ [12, 148, 160, 165, 177, 178, 179, 180, 186]. This gap of roughly one to two orders of magnitude directly reflects the difference in nitrogen activation difficulty and interfacial mass transfer behavior between the two systems. Notably, the NO_3_^−^ + CO_2_ system has already produced several data points approaching the industrial‐grade current density benchmark (−100 mA cm^−2^), whereas the N_2_ + CO_2_ system remains far from this threshold. This visual comparison reinforces the conclusion that the two systems are at different stages of technological maturity: the former is transitioning toward application validation, while the latter remains at the stage of mechanistic exploration and early‐stage performance breakthroughs. Moreover, Figure 15 also illustrates the data comparability issue discussed in this section. Within each system, the current densities vary by more than one order of magnitude, and the FE values show no simple monotonic correlation with current density [17, 52, 64, 81, 160, 178]. This reinforces the argument that performance comparison should focus on ranges and structural patterns rather than on absolute numerical ranking.

**FIGURE 15:**
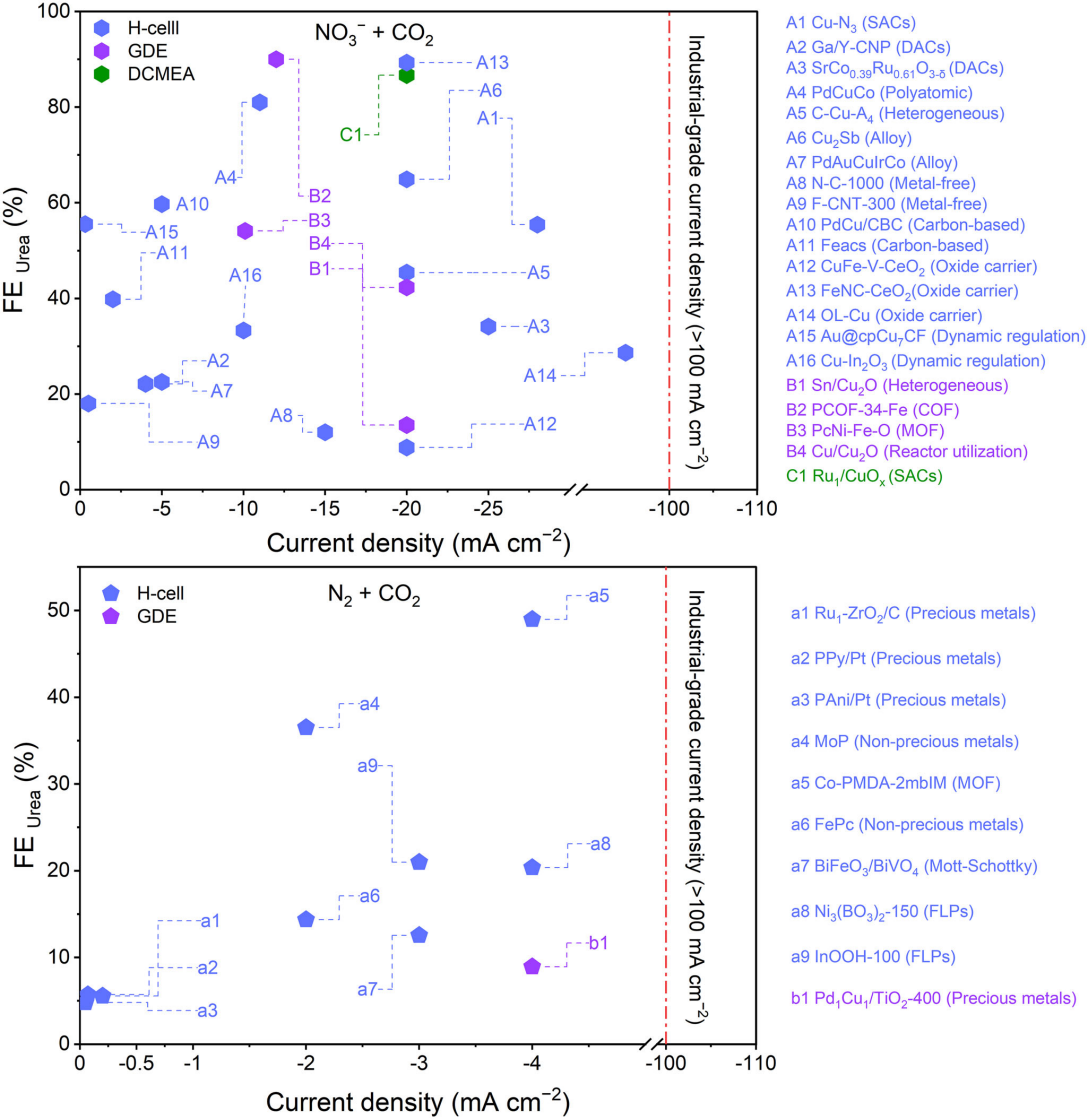
Comparison of urea Faradaic efficiency (FE) versus bias current density for representative NO_3_^−^ + CO_2_ (top) and N_2_ + CO_2_ (bottom) electrocatalytic systems. Different colors indicate reactor types: H‐cell (blue), GDE (purple), and DCMEA (green). The dashed red line marks −100 mA cm^−2^, a benchmark for industrial‐grade current density. The *x*‐axis is broken to highlight the low‐current‐density region while preserving the distance to the industrial benchmark.

From the existing results, the NO_3_^−^ + CO_2_ system has already formed a relatively clear performance stratification pattern: efficient systems typically combine multi‐site synergy, interfacial electronic reconstruction, moderate oxygen vacancies, or confined pore environments, and are progressively advancing toward high current densities and long‐term stable operation [212, 216, 230]. In contrast, although the N_2_ + CO_2_ system has also seen the emergence of high‐potential systems such as conductive MOFs, FLPs, and certain noble‐metal‐like sites, it remains overall at the stage of “demonstrating feasibility → enhancing credibility → gradually improving efficiency.” [12, 186] Particularly in N_2_ electroreduction‐related studies, issues such as nitrogen contamination, background ammonia/urea interference, and insufficient isotopic evidence have long persisted, necessitating more cautious judgment of the performance of the N_2_+CO_2_ system [200]. In other words, the true core task of this system at the current stage is not only “to achieve higher numerical values,” but also “to solidify the evidence, clarify the pathways, and strengthen the reproducibility.”

Therefore, from the perspective of strict technological readiness, the NO_3_^−^ + CO_2_ system has already entered the stage of “transitioning from high‐performance laboratory results to application validation,” while the N_2_ + CO_2_ system remains at the stage of “high‐quality mechanistic exploration in parallel with early‐stage performance breakthroughs.” The two systems are not at the same level of technological maturity, and it is unrealistic to impose the same industrialization expectations on both.

### 4.5 Downstream Separation and Techno‐Economic Perspective

Beyond the electrochemical step, the downstream separation of urea from the dilute aqueous electrolyte remains a major engineering challenge that is often overlooked in laboratory studies. In typical electrocatalytic urea synthesis, the urea concentration is only on the order of millimoles per liter or even lower, while the electrolyte contains a large excess of unreacted NO_3_^−^, supporting electrolyte (e.g., KHCO_3_), and byproducts such as NH_3_ and formate [2, 231]. Separating urea from such a complex dilute solution would require energy‐intensive processes such as evaporation, membrane separation, electrodialysis, or selective crystallization. The choice of separation method strongly depends on the urea concentration, the concentration of interfering ions, and the complexity of the product mixture [8, 84].

A rough estimate of the separation energy can be made by considering the energy required to evaporate water from the electrolyte. The latent heat of water evaporation is approximately 2.26 MJ kg^−1^. For a urea concentration of ∼0.1 M (i.e., ∼6 g urea per liter), removing 1 kg of water would yield only ∼0.6 g urea, corresponding to an evaporation energy of ∼3.8 MJ per kg urea. If the urea concentration drops to ∼10 mM (∼0.6 g L^−1^), the water to be removed per kg urea increases by an order of magnitude, and the evaporation energy rises to ∼38 MJ per kg urea. At even lower concentrations (e.g., ∼1 mM), the separation energy would exceed several hundred MJ per kg urea. These values are already comparable to or even higher than the energy consumption of the conventional Haber–Bosch + Bosch–Meiser process (∼20–30 MJ kg^−1^ urea), highlighting the importance of developing low‐energy separation strategies [6, 7].

Several strategies may help reduce the downstream separation energy. First, increasing the urea concentration in the electrolyte by optimizing the reactor design and improving the FE/yield rate would directly reduce the water‐evaporation energy per kg urea. Second, membrane separation and electrodialysis can selectively enrich urea while rejecting supporting electrolytes and byproducts, although their applicability depends on membrane selectivity, fouling resistance, and long‐term stability [8, 84]. Third, selective crystallization or precipitation of urea from the electrolyte may offer a lower‐energy alternative if the solubility difference between urea and the interfering ions is sufficiently large [231]. Finally, the use of solid‐state electrolytes or membrane electrode assemblies (MEAs) may enable continuous product collection and reduce the volume of dilute electrolyte that needs to be treated [2, 150].

In summary, the overall energy efficiency and economic viability of electrocatalytic urea synthesis depend not only on the FE and yield rate but also on the development of low‐energy downstream separation strategies. This aspect deserves more attention in future work, especially for the N_2_ + CO_2_ system, where the urea concentration is typically much lower than that of the NO_3_^−^ + CO_2_ system.

To further evaluate the engineering relevance of electrocatalytic urea synthesis, a preliminary techno‐economic analysis (TEA) is helpful. Although a full TEA is beyond the scope of this review, a simplified estimation based on electricity consumption can already reveal the key cost drivers and the gap between current laboratory performance and industrial requirements [2, 231].

The electricity consumption per ton of urea can be estimated as:

(5) $$E=\frac{n\times F\times V_{\mathrm{cell}}}{M_{\mathrm{urea}}\times \mathrm{FE}}$$

where n is the number of electrons transferred per urea molecule (16 for NO_3_^−^ + CO_2_ and 6 for N_2_ + CO_2_ under the ideal pathway), F is the Faraday constant (96485 C mol^−1^), V_cell_ is the cell voltage, M_urea_ is the molecular weight of urea (60.06 g mol^−1^), and FE is the Faradaic efficiency. Assuming an electricity price of 0.03–0.05 USD kWh^−1^, a cell voltage of 2–4 V, and a urea FE of 30–90%, the electricity cost for urea production is estimated to be on the order of several hundred to over one thousand USD per ton of urea [2, 8]. For comparison, the current market price of urea is approximately 300–500 USD per ton [6], indicating that the electricity cost alone may already exceed the product value under typical laboratory conditions.

The situation is more favorable for the NO_3_^−^ + CO_2_ system, where several catalysts have achieved FE above 80% and urea yield rates on the order of hundreds of mmol h^−1^ g_cat_^−1^ [84]. If the FE can be maintained above 80% at industrial current densities (> 100 mA cm^−2^) and the electricity price is low (e.g., < 0.03 USD kWh^−1^), the electricity cost could drop to a few hundred USD per ton of urea, making the route potentially competitive, especially when coupled with wastewater treatment and CO_2_ utilization [7, 33]. In contrast, the N_2_ + CO_2_ system currently suffers from much lower FE and yield rates, and its electricity cost is far from economically viable [12, 84].

It should also be noted that the downstream separation cost discussed above may dominate the overall cost. For a dilute urea solution (∼10 mM), the separation energy alone (tens of MJ per kg urea) could translate into an additional cost comparable to or even higher than the electricity cost of the electrochemical step [33, 221]. Therefore, the economic viability of electrocatalytic urea synthesis depends on the synergistic optimization of catalyst activity, selectivity, reactor design, and downstream separation. Future TEA studies should integrate these factors into a complete process model to provide more reliable guidance for scale‐up [33, 231].

### 4.6 Application Suitability Scenarios and Development Priorities

Based on the above comparison, the positioning of the two systems in future applications can be more clearly defined. The most practically significant application scenario for the NO_3_^−^ + CO_2_ system is a coupled process that integrates nitrate‐containing wastewater treatment with CO_2_ utilization from flue gas or industrial tail gas [84, 232]. For scenarios such as chemical, electroplating, metallurgical, agricultural runoff, and certain domestic wastewater treatment, this system not only enables pollutant removal but also converts both nitrogen and carbon into high‐value‐added products, exhibiting the dual attributes of “treating waste with waste” and resource recovery [233, 234]. Especially as catalyst stability, reactor durability, and product separation strategies further mature, this route possesses strong potential for deployment in distributed wastewater treatment units. In other words, the application value of the NO_3_^−^ + CO_2_ system is not merely reflected in urea itself, but also in its capacity to transform environmental remediation processes into value‐creation processes.

The N_2_ + CO_2_ system, on the other hand, is more suitable for deployment as a long‐term strategic direction. Its ideal vision is to directly utilize N_2_ from the air and CO_2_ from industrial emissions or air capture, driven by renewable electricity, to synthesize urea under ambient temperature and pressure, theoretically completely breaking free from the reliance of the conventional Haber–Bosch/urea two‐step process on fossil fuels and centralized large‐scale facilities [207]. Once this route achieves substantial breakthroughs in N_2_ activation efficiency, coupling selectivity, and device scale‐up, it will have disruptive significance for distributed green chemical production in agricultural regions, remote areas, offshore platforms, or regions rich in renewable energy. However, based on the current foundation, this goal still requires solving a series of fundamental issues, including highly credible evidence of N_2_ activation, stable and reproducible efficient catalysts, reactor systems suitable for dual‐gas feeding, as well as continuous urea separation and system energy efficiency evaluation. Therefore, a more reasonable development strategy is not to view it as a short‐term alternative route, but rather to position it as a long‐term strategic direction for future green chemical systems [207, 208].

From a temporal perspective, the two systems can be considered to correspond to different development priorities. In the next 1–5 years, the NO_3_^−^ + CO_2_ system holds greater promise for taking the lead in completing pilot‐scale demonstration and scenario validation. Over the next 5–10 years or even longer, the N_2_ + CO_2_ system will still primarily undertake the tasks of scientific frontier breakthroughs and fundamental technological reserve. Once the latter achieves breakthroughs in key bottlenecks, its strategic significance may far surpass that of the former. However, at the current stage, prioritizing the industrial validation of the NO_3_^−^ + CO_2_ system is clearly more aligned with technological maturity and resource investment efficiency.

## 5 Summary

Electrocatalytic urea synthesis, as an important technological pathway connecting the carbon and nitrogen cycles, demonstrates broad prospects in achieving low‐carbon chemical industry and resource recycling. This review has systematically summarized the NO_3_^−^ + CO_2_ and N_2_ + CO_2_ systems from the aspects of feedstock characteristics, catalyst design, reaction mechanisms, performance optimization, and engineering exploration, while also conducting a comparative analysis of the essential differences and development positioning of the two systems.

Overall, the NO_3_^−^ + CO_2_ system, by virtue of its advantages including facile nitrogen source activation, high aqueous accessibility, and abundant intermediate generation pathways, has achieved significant progress in Faradaic efficiency, urea yield rate, and reactor compatibility, with some systems attaining FE approaching 90% and yield rates on the order of hundreds of mmol h^−1^ g_cat_^−1^, demonstrating promising potential for engineering applications. Its catalyst design has gradually shifted from single active site optimization to multi‐site synergy, interfacial electronic structure modulation, and reactor intensification coupling, reflecting a development trend from “performance enhancement” toward “system optimization.” In contrast, although the N_2_+CO_2_ system holds higher theoretical appeal in terms of feedstock availability and sustainability, it remains significantly lagging in overall performance due to constraints such as the high energy barrier of the N≡N bond, low solubility, and difficulties in intermediate stabilization. Current research efforts are concentrated on enhancing N_2_ activation, stabilizing key intermediates, and optimizing C–N coupling pathways, still remaining at a stage where mechanistic exploration and performance improvement proceed in parallel.

Through the systematic comparison of the two systems, it can be found that their differences are not only reflected at the level of performance metrics, but more fundamentally in the reaction essence and design logic. The core of the NO_3_^−^ + CO_2_ system lies in the kinetic matching between NO_3_^−^RR and CO_2_RR and the synergistic regulation of multiple intermediates, whereas the N_2_ + CO_2_ system is premised on N_2_ activation, emphasizing the effective activation of inert molecules by active sites and the sustained supply of intermediates. This fundamental difference determines the significant divergence between the two systems in terms of catalyst design strategies, reactor requirements, and engineering implementation pathways, and further clarifies their dual‐track development pattern of “near‐term deployable route” and “long‐term strategic goal route.”

Looking forward, the development of electrocatalytic urea synthesis should place greater emphasis on the synergistic optimization of catalyst design and reactor engineering, while integrating renewable energy supply to achieve the transition from “high‐performance laboratory results” to “practical application feasibility.” In terms of development pathways, the NO_3_^−^ + CO_2_ system is expected to take the lead in realizing pollutant resource utilization and pilot‐scale validation in the short term, while the N_2_ + CO_2_ system requires continued breakthroughs in fundamental mechanisms and key technologies, gradually advancing toward a truly green distributed urea synthesis paradigm. The synergistic advancement of both systems will provide important support for the construction of a low‐carbon, efficient, and sustainable future chemical industry.

## Author Contributions

**Fulin Dong**: conceptualization, investigation, visualization, writing – original draft. **Ruihan Zhang**: resources, validation. **Huiyu Lou**: resources, validation. **Tianshi Gao**: resources, validation. **Guangjin Zhang**: supervision, writing – review and editing. **Menglei Yuan**: supervision, funding acquisition, project administration, writing – review and editing.

## Conflicts of Interest

The authors declare no conflicts of interest.

## Data Availability

Data sharing not applicable to this article as no datasets were generated or analysed during the current study.
